# Blueprint of a smokescreen: Introducing the validated climate disinformation corpus for behavioural research on combating climate disinformation

**DOI:** 10.1111/bjop.70012

**Published:** 2025-07-18

**Authors:** Tobia Spampatti, Tobias Brosch, Christian Mumenthaler, Ulf J. J. Hahnel

**Affiliations:** ^1^ Center for Conflict and Cooperation and Department of Psychology New York University New York City New York USA; ^2^ Department of Psychology and Swiss Center for Affective Sciences University of Geneva Geneva Switzerland; ^3^ University of Applied Sciences and Arts Western Switzerland (HES‐SO) Carouge Switzerland; ^4^ Faculty of Psychology University of Basel Basel Switzerland; ^5^ Faculty of Sustainability Leuphana University Lüneburg Lüneburg Germany

**Keywords:** climate disinformation, misinformation, social media

## Abstract

Behavioural science research has the potential to develop evidence‐based strategies to fight disinformation about climate science and climate mitigation action; however, this research has yet to be conducted systematically with validated sets of climate disinformation stimuli. Here, we present the Climate Disinformation Corpus, a collection of climate disinformation statements designed to systematize experimental research testing future disinformation interventions. Using computational social science techniques, we gathered climate disinformation stimuli from the social media platform Twitter/X. We identified 78 statements containing disinformation about the existence, the causes, the consequences of climate change, the reliability and objectivity of climate scientists, and arguing for the delay of climate policies. The Climate Disinformation Corpus showed good heterogeneity across 15 validation measures (e.g., perceived persuasiveness, perceived trustworthiness, and sharing intentions) in a validation study involving a representative sample of *N* = 503 British participants. Furthermore, the climate disinformation statements were correlated with four individual differences measures related to belief in climate science and support for climate actions, congruently with theoretical expectations. We conclude with practical suggestions on implementing the Climate Disinformation Corpus in disinformation research according to different research questions.

## BACKGROUND

According to the latest reports, the world is heading into more than 2.7°C of warming from preindustrial levels without immediate action (The CAT Thermometer, 2022; IPCC, 2021). This looming climate crisis represents unprecedented risks of loss of ecosystems (IPCC AR6 WGII, 2022), health hazards (Atwoli et al., 2021; Mogwitz et al., 2022; UNECE, 2022) such as extreme heat and natural disasters (Thomas et al., 2021), and geopolitical instability projected to displace millions (Cattaneo et al., 2019; Miranda et al., 2023). More urgent actions are needed to mitigate climate change (Smith et al., 2019; Höhne et al., 2020). It is therefore surprising and alarming that climate change mitigation has been insufficient and more discursive than systemic (Biermann et al., 2022; United Nations Environmental Programme, 2024).

The causes of societal inaction are multifaceted (Brulle & Norgaard, 2019; Hornsey & Lewandowsky, 2022; Martinez‐Alvarez et al., 2022), but one identified driver is disinformation spread by the climate change countermovement (Dunlap & Brulle, 2020; House Oversight Committee, 2022; IPCC AR6 WGII, 2022, 1939; Kornek et al., 2020; Nyhan et al., 2022; Oreskes & Conway, 2010; Spampatti, Hahnel, et al., 2023; Winter et al., 2024). This top‐down spread of climate disinformation from vested interests to policymakers and the populace falls into two macro‐categories (Kinol et al., 2025): *intentionally* raising doubts about climate science (Coan et al., 2021) and delaying climate actions and policies (Lamb et al., 2020). Disinformation about climate science refers to the ill‐intentioned dismissal of the accumulated scientific evidence about anthropogenic climate change (see Cook, 2020). Previous studies have catalogued more than 50 subtypes of climate disinformation, which target the existence, the causes, the consequences of climate change or the efficacy of mitigation policies, or the reliability and objectivity of climate scientists (Coan et al., 2021). For example, a declaration, signed by two Nobel laureates misleadingly, claims that climate models are wrong, and is being used to question the scientific consensus (https://clintel.org/world‐climate‐declaration/). Arguments to delay climate actions and policies are a more recent, policy‐focused communication strategy that aims at setting (or stalling) the political agenda by antagonizing the actions to be taken and misrepresenting who is responsible for it, their timeline, and the distribution of their costs and benefits (Lamb et al., 2020). Recent literature documented how vested interests manipulate legitimate concerns with four subtypes of argumentation for delaying climate action: redirecting and minimizing responsibility, pushing non‐transformative solutions, emphasizing the downsides of climate actions, or surrendering to the consequences of climate change. For example, artificial intelligence industries argue for blindly trusting technological progress to solve the energy crises of today to avoid having to reduce current and future resource consumption (Crawford, 2024; Hao, 2024).

Disinformation about climate science and arguments to delay climate policies can be considered disinformation^1^ because of actors *intentionally* weaponizing legitimate public concerns and/or presenting information to misleadingly engender doubt about the scientific consensus of the causes and consequences of climate change (House Oversight Committee, 2022; Lamb et al., 2020; Lewandowsky et al., 2016; Wardle & Derakhshan, 2017). This can lead to societal inaction and to public harm, as it directly threatens democratic political and policymaking processes and indirectly threatens public goods such as the environment (European Commission, 2018). One result of the top‐down spread of disinformation in the past decades is that up to a third of the global population doubts that human activities are causing climate change and that its consequences will affect them (Leiserowitz et al., 2023).

There are multiple explanations for why people are susceptible to climate disinformation (Chen et al., 2021; Ecker et al., 2022; Pennycook & Rand, 2021; Philipp‐Muller et al., 2022; Spampatti, Hahnel, et al., 2023; Van Bavel et al., 2021). The account that has received the most support to date is the *partisanship bias hypothesis* (Doell et al., 2021; Van Bavel & Pereira, 2018; Van Bavel et al., 2024). This framework argues that when disinformation is concordant with people's political ideology or has become internalized in people's political identity, disinformation is more readily accepted and shared. This partisan bias can be due to selective exposure to partisan news or to motivated reasoning. These processes override people's motivation to hold accurate beliefs and spread accurate information (Van Bavel et al., 2021; e.g., Pretus et al., 2023). Cross‐cultural evidence finds that agreement with disinformation about climate science is a facet of modern conservative (economic and social) ideology and identity (Berkebile‐Weinberg et al., 2024; Doell et al., 2021; Hornsey et al., 2018), mostly due to a high prevalence of messaging from the climate change countermovement in conservative news (Feldman et al., 2012; Oreskes & Conway, 2010). Moreover, two recent model comparison studies find that a partisanship bias model overperforms other models (e.g., Pennycook et al., 2022) in accounting for people's susceptibility to believing misinformation in general and disinformation about climate change in particular (Borukhson et al., 2022; Spampatti, Hahnel, & Brosch, 2024). Overall, the partisanship bias suggests that people will believe and share disinformation about climate science and arguments to delay climate action more when they are internalized in their political ideology and identity.

Behavioural scientists are researching strategies to combat climate disinformation and climate inaction (Cologna & Oreskes, 2022). Behavioural interventions against climate disinformation are mainly summarized as fact‐checking and psychological inoculations, based on whether they are employed before or after vested interests spread climate disinformation (Lewandowsky, 2021; Lewandowsky & van der Linden, 2021; Schmid & Betsch, 2019; but see Cook et al., 2023; for a toolbox of interventions against misinformation on topics such as politics or health behaviours, see Kozyreva et al., 2024). Fact‐checks (debunking) are corrections containing factual information about climate science or action, distributed after countermovement actors propagate disinformation (Porter et al., 2019; Porter & Wood, 2021; Walter & Murphy, 2018). Psychological inoculations (prebunking) are messages providing psychological resources to resist disinformation before people encounter them (Cook et al., 2017; Green et al., 2022; Spampatti, Brosch, et al., 2024; Spampatti, Hahnel, et al., 2023; van der Linden et al., 2017; Vraga et al., 2019, 2020). Overall, debunking and prebunking can have a positive effect on beliefs (Banas & Rains, 2010; Lu et al., 2023; Walter et al., 2020; but see Chan & Albarracín, 2023; Modirrousta‐Galian et al., 2023), and partnerships of academics with industry have begun implementing these strategies in the field (Roozenbeek, Maertens, et al., 2022; Roozenbeek, van der Linden, et al., 2022; Spampatti, Brosch, et al., 2023).

Although the results of experimental research on combating disinformation are encouraging, to our knowledge, research on climate disinformation has yet to be conducted with stimuli that have been validated in previous or independent studies. Instead, experimental stimuli that mimic communication from the climate change countermovement have consisted of *ad hoc* disinformation, either selected from real disinformation but without theoretical justification (Green et al., 2022; Young et al., 2018), created anew by researchers (McCright et al., 2016), or presented without explanation (Chockalingam et al., 2021; Cook et al., 2017; Vraga et al., 2020). Moreover, these stimuli have rarely been pretested (but see Spampatti, Hahnel, et al., 2023; van der Linden et al., 2017; Winter et al., 2022). Deliberately selecting validated disinformation is a critical step for viable experimental designs and influences the effect size of an intervention (Drost, 2011; Maertens, 2022; Pennycook et al., 2021). Study results based on single, unvalidated stimuli run a high risk of failing to generalize outside the specific instance of investigation, a phenomenon known as the fixed‐effect fallacy (Clark, 1973). As Yarkoni (2022) notes, failing to use a large set of validated stimuli can threaten generalizability and lead to false‐positives or inconsistent effect sizes and to spurious statistically significant effects that are misattributed to constructs of interest when they are due to unaccounted stimuli variation in single studies. This problem was recently addressed in the general misinformation research with the development and validation of a domain‐general measure of susceptibility to misinformation (i.e., the Misinformation Susceptibility Task, Maertens et al., 2024). However, no such approach exists for climate disinformation research, which risks becoming non‐comparable and its foundations fragile.

To illustrate the problem, consider the ‘backfire effect’ (Nyhan & Reifler, 2010). This effect suggests that, when corrected, some people may believe *more* in the incorrect belief, the correction thus ‘backfiring’. The original finding has over 3900 citations (Google Scholar, April 2025) and captivated academics and media (Nyhan, 2021). However, follow‐up research did not replicate this effect, neither with the original stimuli nor across a larger number of topics (Guess & Coppock, 2020; Haglin, 2017; Wood & Porter, 2019). Swire‐Thompson et al. (2020) contended that the backfire effect is found exclusively in studies where measurement validity and reliability are low and concluded that backfire concerns are overblown (Ecker et al., 2023; Swire‐Thompson et al., 2022). While the correction‐resistant belief in the backfire effect is an internal joke of the disinformation research community,^2^ the example stresses how important validated and heterogeneous stimuli and measures are to systematically investigate disinformation and behavioural interventions to counteract it, to provide reliable and actionable conclusions (Flake & Fried, 2020; Yarkoni, 2022).

*Ad hoc* selection of disinformation as experimental stimuli may be justified when there is a need for developing specific interventions in new (dis)information scenarios (Maxmen & Mallapaty, 2021). However, the decades‐long history of the climate change countermovement (Michaels, 2020; Oreskes & Conway, 2010) and the facets of their disinformation activity are well documented (Coan et al., 2021; Cook, 2020; Jacquet, 2022; Lamb et al., 2020; Supran & Oreskes, 2021). This provides theoretical justifications for identifying specific and multiple types of climate disinformation to research behavioural disinformation interventions. Choosing or creating *ad hoc* disinformation might focus resources on fighting disinformation subtypes that do not threaten climate policy support. As a symptom of this problem, a recent meta‐analysis found no evidence that fact‐checking scientific misinformation is effective and that the research suffers from great unaccounted heterogeneity (Chan & Albarracín, 2023); a recent registered report furthermore found a limited effect of psychological inoculations against multiple climate disinformation (Spampatti, Hahnel, et al., 2023). Overall, research on behavioural disinformation interventions must systematically consider the heterogeneity of the different climate disinformation types to provide reliable and generalizable disinformation interventions.

Therefore, the current study aims to advance experimental practices in disinformation research (Camargo & Simon, 2022; Lin et al., 2023; Pennycook et al., 2021; Roozenbeek et al., 2024; Roozenbeek, Maertens, et al., 2022; Roozenbeek, van der Linden, et al., 2022; Spampatti, 2024) with a validated set of climate disinformation statements to use as stimuli mimicking communication from the climate change countermovement in future misinformation and disinformation research. We systematically collected real posts spread by public figures of the climate change countermovement on the social media platform Twitter (before it was rebranded ‘X’; Effrosynidis et al., 2022; Falkenberg et al., 2022; Fownes et al., 2018; Hopke & Hestres, 2018; Meyer et al., 2023; Mumenthaler et al., 2021). We sorted the posts about climate science and climate mitigation action according to two classifications of climate disinformation (Coan et al., 2021; Lamb et al., 2020) to then measure how a representative sample of British participants perceived the climate disinformation across 13 validation measures that are the current gold standard to rigorously evaluate misinformation stimuli (Pennycook et al., 2021). We complemented these measures by asking participants how disinformation made them feel about climate science and action, as affective reactions are precursors and motivators of policy support and are therefore discriminant dimensions for evaluating experimental stimuli (Lodge & Taber, 2013; Slovic & Peters, 2006). We expected the disinformation to make participants feel negatively towards climate science and climate mitigation actions. These validation measures were selected as they are relevant characteristics of (mis)information stimuli (e.g., perceived familiarity increases the influence of misinformation; see Pillai & Fazio, 2021) and are frequently assessed in misinformation studies (and thus researchers may be interested in pretested values; Pennycook et al., 2021). Following guidelines for conducting misinformation research (Pennycook et al., 2021), we furthermore tested the association between the validation measures and individual differences that are established predictors of climate scepticism (Hornsey et al., 2018; Hornsey & Lewandowsky, 2022; Jylhä et al., 2023; Van Bavel et al., 2021): political ideology (Hornsey et al., 2016; Pretus et al., 2023; Spampatti, Hahnel, & Brosch, 2024), trust in scientists (Cologna & Siegrist, 2020; Safford et al., 2020), climate change opinions (Leiserowitz et al., 2021), and tendency for deliberate thinking (Ali & Qazi, 2022; Pennycook et al., 2022). We expected climate disinformation to paint a negative picture of climate science and action and for individual differences to be associated with the perceived persuasiveness of disinformation. The Climate Disinformation Corpus thus developed will enable researchers to systematically examine the direct influence of climate disinformation on public opinion, test new evidence‐based interventions to fight climate disinformation with a validated and standardized set of stimuli, and compare the effectiveness of interventions.

## METHODS

**FIGURE 1:**
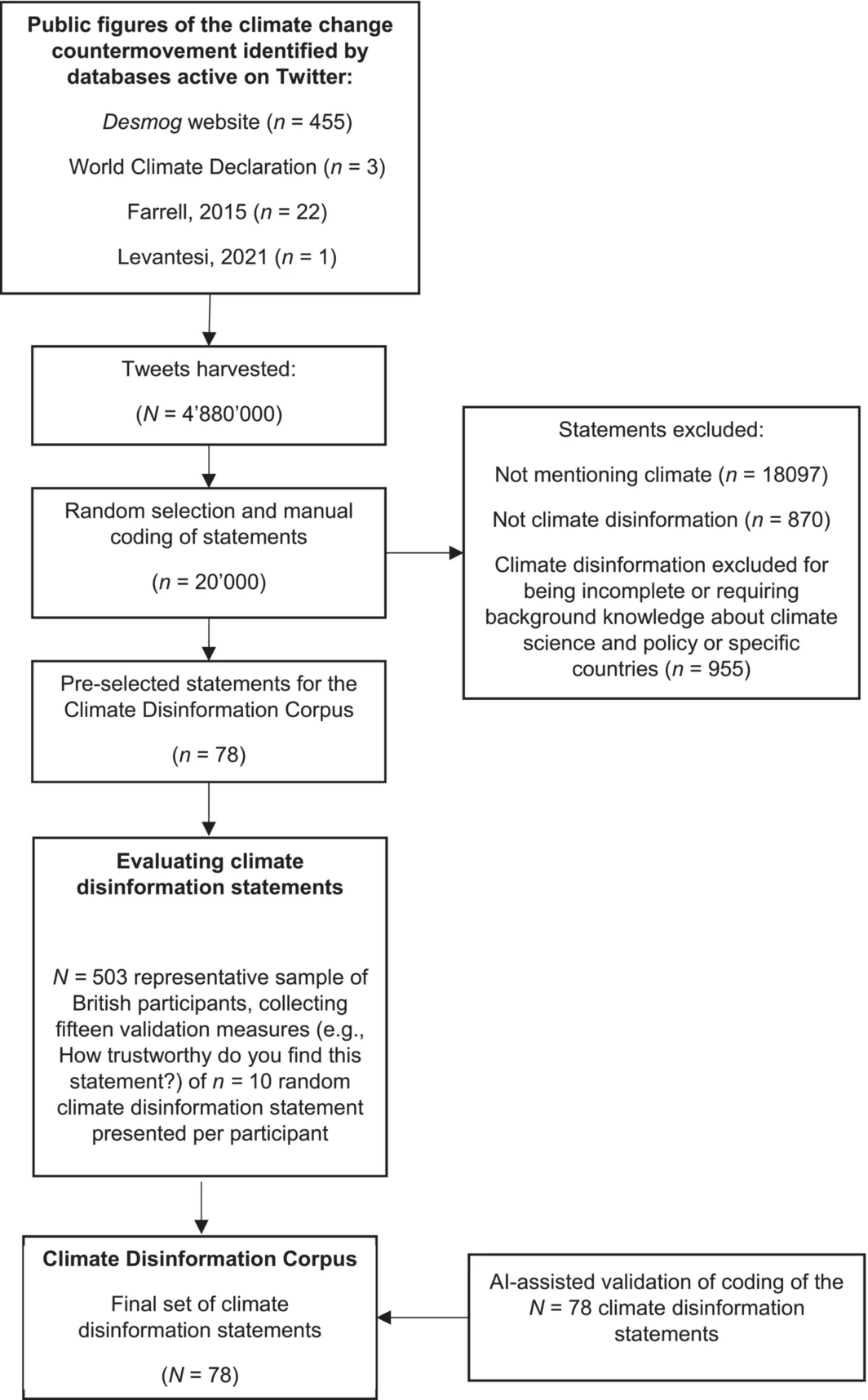
Explanatory diagram, identification of climate disinformation statements, and validation study. Those public figures of the climate change countermovement that have been identified by multiple sources are counted in the *Desmog* database.

This study was originally conducted to validate experimental stimuli for a cross‐cultural registered report that tested the effectiveness of six misinformation interventions against a subset of these climate disinformation statements (Spampatti, Hahnel, et al., 2023). Data, materials, and code are available at: https://osf.io/prxtn/?view_only=85a19bf047be4abf81beb59282d2d287. Due to the nature of the data, the validation results of the Climate Disinformation Corpus^3^ are available online only for scientific use https://doi.org/10.23668/psycharchives.13465 (see https://psycharchives.org/en/about#sharing_levels). The University Commission for Ethical Research in Geneva (CUREG2.0) of the University of Geneva, Switzerland (ID: CUREG‐2022‐05‐43‐Corr‐Brosch) approved the Twitter data collection and the validation study. Participants consented to the study and received £2.25 for participation. A study breakdown is presented in Figure 1.

### Sample size and justification

We collected a representative sample of *N =* 503 British Prolific users (*n =* 253 females, mean_AGE_ = 46.73 ± 29.62 years) based on two considerations. First, we mirrored the sample size Pennycook et al. (2021) used in their validation guidelines, to ensure that each climate disinformation statement received between 30 and 70 ratings (mean_*N* ratings_ = 63.58 ± 10.26; min_*N* ratings_ = 36, max_*N* ratings_ = 113). Second, since political ideology is a main precursor of (dis)believing climate change (Hornsey et al., 2016), we opted for British participants over other nationalities, as the effect of political ideology on climate beliefs in the UK is most similar to the overall effect observed across 25 countries (Hornsey et al., 2018; see also Berkebile‐Weinberg et al., 2024). We thus aimed to preserve the influence of political ideology on the ratings of disinformation statements in a manner that would apply in the UK and beyond.

### Online survey procedure

Participants accessed the Qualtrics survey online by link on the Prolific website.^4^ After consenting, participants' demographics (gender, age, education) were collected, as were their trust in scientists, economic and social political ideology (see Materials), and social media presence (*Do you have an active social media account?* [Yes/no]; Pennycook et al., 2021). Participants subsequently responded, in random order, to two individual difference measures related to climate disinformation susceptibility: their opinion on climate change, assessed with the Six Americas Super Short Survey (Chryst et al., 2018); and their tendency for deliberate thinking, assessed with the Cognitive Reflection Task, Version 2 (Thomson & Oppenheimer, 2016). We embedded a two‐strikes‐out attention check within this section: participants responded to a soft attention check (*Please select ‘3’ to show you are paying attention*), resulting in a warning with a 10‐s penalty upon failure. Inattentive participants were then presented with the same attention check, which would have removed them if they provided a second inattentive answer. As no participant failed this attention check, all participants randomly received 10 of 80 climate disinformation statements^5^ as text‐based stimuli, responding, for each item, to 15 validation measures drawn from guidelines for validating disinformation material (e.g., perceived persuasiveness, perceived manipulativeness, sharing intentions; Pennycook et al., 2021), their presentation order randomized between participants. The survey concluded with the debriefing, where we highlighted the misleading nature of the statements. The debriefing also contained a treatment intervention, as a message about the scientific consensus behind anthropogenic climate change. Consensus messaging is an established intervention that lowers scientific misperceptions about climate change (van Stekelenburg et al., 2022; Većkalov et al., 2024), which we used to mitigate the effects of reading the climate disinformation statements. The debrief was time‐locked to 5 s to force participants to read it (see Data S1). The survey lasted 15 min.

## MATERIALS

### Identification of climate disinformation statements

We identified the climate disinformation statements using a two‐step procedure. First, we compiled a list of public figures of the climate change countermovement with an active Twitter account. Public figures were identified by academic and journalistic resources that provided evidence of these actors spreading climate disinformation and/or being funded by vested interests that profit from fossil fuel production (Farrell, 2016; Levantesi, 2021; https://www.desmog.com/climate‐disinformation‐database/). Moreover, we included the Twitter‐active signatories – at the time of data collection – of the disinformation document ‘World climate declaration’ (https://clintel.org/world‐climate‐declaration/) that misleadingly claims that climate change is not real and is being used to question the scientific consensus.

With the Twitter API, we then collected 4,880,000 tweets by the climate change countermovement, from account creation until April 2022, and then from the week leading to and the two weeks of the UNFCCC Conference Of the Parties 27 (COP 27, 31 October–20 November 2022), a high‐profile international climate event with a significant online presence of related disinformation (Climate Action Against Disinformation, 2023; Meyer et al., 2023). Whereas previous computational social science approaches used keywords (e.g., ‘climate change’) or hashtags (e.g., ‘#globalwarming’) as search terms (Chen et al., 2022; Effrosynidis et al., 2022; Meyer et al., 2023; Yeo et al., 2017), we selected this approach to map the arguments used to disinform the public about climate change from top‐down, by main public figures. Data collection thus mirrored how climate disinformation ecologically spreads from vested interest into public discourse (Dunlap & Brulle, 2020; Farrell, 2016; IPCC AR6 WGII, 2022; Oreskes & Conway, 2010). Rather than missing social media posts that spread climate disinformation without using hashtags, this data collection approach furthermore enabled us to comprehensively collect all potentially disinformation statements spread by the climate change countermovement.

Second, we randomly selected *n =* 10,000 tweets from each data collection (account creation – April 2022, and COP 27, 31st of October–20th of November 2022), *N =* 20,000 tweets in total, which the first author manually coded according to their relatedness to climate change (1 = *not at all related to climate change* to 4 = *absolutely related to climate change*), their level of disinformation (1 = *not at all disinformation about climate change* to 4 = *absolutely disinformation about climate change*) and argumentation delaying climate action (1 = *not at all a delay argument about climate change policies* to 4 = *absolutely a delay argument about climate change policies*). The coder matched the statements with the disinformation categories by Coan et al. (2021) and by the arguments of climate delay by Lamb et al. (2020). For example, Statement 10, ‘*No sane and or honest person believes climate change or CO2 emissions are an existential threat to humanity. Not one*’, was categorized as disinformation about climate impacts not being bad according to the definition provided by Coan et al. (2021): ‘*Disinformation questioning the consequences of climate change. Can contain claims such as* “*CO*_*2*_ *is not a pollutant*” *and* “*warming of only a few degrees*”’ (page 10). Readers should note that, because statements belonging to both categorizations can be *intentionally* weaponized to misleadingly public opinion and cause public harm (European Commission, 2018), we hereafter refer to the statements belonging to any combination of these two classifications as ‘climate disinformation statements’.

We identified *n* = 1033 statements as containing disinformation about climate science and/or delay arguments against climate action.^6^ Out of this initial set of statements, we identified *N* = 78 climate disinformation statements (*n* = 38 of the five types of disinformation about climate science and *n* = 40 of the four types of arguments to delay climate action^6^; see Table S1 for a breakdown) that the research team agreed were complete, intelligible statements that would not require participants to attain background knowledge about climate science and policy or specific countries. We then removed all meta‐data, authorship information, URLs, mentions, images, videos, marketing materials, and hashtags. Afterward, statements were again checked for comprehensibility. Examples of climate disinformation statements are in Table 1; the full item list is available in Table S2.

**TABLE 1:** Example climate disinformation statements in the Climate Disinformation Corpus.

Disinformation type	Text
Disinformation about climate science	The French Alps are snow capped in early November (just as they always have been). How can that be if CC is warming the Earth? A thinking person just might see that the whole CC thing is complete BS. Little warming with little change in climate. Relax everything is normal
Disinformation about climate science	The evidence for manmade climate change is so thin they cannot debate it. They hide behind the lie of consensus. There is no room for consensus in science. The basis is a provable hypothesis. There is not a single peer reviewed study that proves manmade CO_2_ is causing global warming
Delay argument against climate action	Fossil fuels have helped the majority of the 8 billion people in the world to have much better lives. Over half of food production is dependent on fossil fuel derived fertilizers. The climate crazies will starve people by limiting nitrogen fertilizers
Delay argument against climate action	Net Zero will make us Net Poorer. No one voted to be Net Poorer

As having a single coder identify the 78 climate disinformation statements can introduce subjectivity bias into the coding, we verified the ratings with the ChatNetZero large language model (Version 1.0; https://chatnetzero.ai/; Hsu et al., 2024; see Section S1: Data S1, for a full description of the procedure). Large Language Models have been found to accurately rate different dimensions of textual data, with very high correlations between their ratings and human ratings (Rathje et al., 2024). Among the different options, ChatNetZero is a fine‐tuned algorithm using a collection of credible reports and data verified by experts from the Net Zero Tracker (https://zerotracker.net/) to generate climate relevant responses that are verified and less prone to AI‐based hallucination (i.e., when a model produces nonsensical or inaccurate outputs; Weise & Metz, 2023). We thus prompted ChatNetZero to provide a rating for the level of disinformation and argumentation delaying climate action for each disinformation statement (*Rate the following statement from 1 = not at all disinformation about climate change to 4 = absolutely disinformation about climate change, and from 1 = not at all a delay argument about climate change policies to 4 = absolutely a delay argument about climate change policies, according to your knowledge*; see Table S2 for the full ratings). All but two of the climate disinformation statements were categorized as either climate disinformation or arguing for the delay of climate actions, which suggests that LLMs trained on accurate scientific information notice the misleading nature of most of the final selection of climate disinformation statements. Because of the limited number of climate disinformation statements making statistical comparisons underpowered, we only provide descriptive statistics of the LLM categorization (cf. Rathje et al., 2024). Overall, the algorithm categorized 64 out of 78 statements as being disinformation about the science of climate change and 64 out of 78 statements as being arguments of climate delay. Only two statements, statement nr. 28 and statement nr. 48, were coded as being somewhat or slightly delay arguments about climate change policies. As a robustness check of the categorization performance of ChatNetZero, we also analysed 30 fact‐check statements about climate change that were predominantly categorized as accurate (see Section 1: Data S1).

### Validation measures

Validation measures were taken from Pennycook and colleagues' guidelines (Pennycook et al. 2021): *How trustworthy do you find this statement?* [1 = not at all to 7 = very] *What is the likelihood that the above statement is true?* [1 = Extremely unlikely to 6 = Extremely likely]; *How favourable would it be to liberals* versus *conservatives?* [1 = More favourable for liberals to 6 = More favourable for conservatives]; *If you were to see the above article on social media, how likely would you be to share it?* [1 = Extremely unlikely to 6 = Extremely likely]; *How exciting [worrying/funny/informative] is this statement?* [1 = Not at all to 6 = Extremely]; *To what extent do you find this statement outrage provoking?* [1 = Not at all to 6 = Extremely]; *Assuming that the statement is entirely accurate, how important would this statement be?* [1 = Extremely unimportant to 6 = Extremely important]; *How manipulative do you find this headline?* [1 = not at all to 7 = very]; How persuasive do you find this headline? [1 = not at all to 7 = very]; *Are you familiar with the above statement (have you seen or heard about it before)?* [Yes/unsure/no]. We assessed how the statements made participants feel towards climate mitigation action and climate science: *How does this statement make you feel about climate mitigation action [climate science]?* [1 = very negatively to 10 = very positively] to measure the impact of the climate disinformation statements on participants' affect towards climate science and actions (Slovic & Peters, 2006). In addition, we collected the engagement metrics (likes and retweets) for each statement (see Table 2).

**TABLE 2:** Descriptive summary statistics of the climate disinformation statements.

	Mean	SD	Median	Scale min	Scale max
Validation measures					
Affect: climate science	5.12	2.35	5	1	10
Affect: climate action	5.07	2.34	5	1	10
Importance	4.25	1.44	4	1	6
Sharing intentions	1.66	1.20	1	1	6
Perceived as being true	2.89	1.57	3	1	6
Political slant	4.15	1.43	4	1	6
Manipulative	4.61	1.86	5	1	7
Persuasive	3.02	1.80	3	1	7
Trustworthiness	3.02	1.69	3	1	7
Excitement	1.27	0.78	1	1	6
Funny	1.30	0.84	1	1	6
Worry	2.85	1.69	3	1	6
Informative	2.27	1.43	2	1	6
Outrage	3.73	1.55	4	1	6
Engagement metrics					
Likes	984.50	2941.32	50	0	21,874
Retweets	387.40	1310.02	26	0	10,659

*Note*: Individual statistics for each climate disinformation statement are available upon request.

### Individual differences measures

We assessed participants' economic and social political ideology with a visual analogue scale (Vlasceanu et al., 2024; 2‐items: *What is your political orientation for the issues listed below? Please note, by ‘liberal’ we mean classically left‐wing, and by ‘conservative’, we mean classically right‐wing*. (1) *For social issues* (e.g., *health care, education*, etc.); (2) *For economic issues* (e.g., *taxes*) [0 = Extremely liberal/left‐wing, 50 = Moderate, 100 = Extremely conservative/right wing]. *n =* 12 participants chose not to respond). We then assessed participants' trust in scientists (single‐item; *How much do you trust scientists to do what is right for your country?* [1 = Not at all; 2 = Not too much; 3 = Some; 4 = A lot]; Pew Research Center, 2020).

We assessed participants' opinions about climate change with the Six Americas Super Short Survey (Chryst et al., 2018). This measure, applicable outside the American context (Leiserowitz et al., 2023), assesses belief in climate change and motivation to act against it with four items that cover respondents' climate change worry, expected harm to future generations and oneself, and personal importance (*How important is the issue of global warming to you personally?* [1 = Not too important to 4 = Extremely]; *How worried are you about global warming?* [1 = Not very to 3 = Very]; *How much do you think global warming will harm you personally?* [0 = Don't know; 1 = Not at all to 4 = A great deal]; *How much do you think global warming will harm future generations of people?* [0 = Don't know; 1 = Not at all to 4 = A great deal]). We initially classified respondents into the six climate opinion groups (Chryst et al., 2018); however, as the algorithm did not classify 34% of the sample, we report each item separately.

We assessed participants' tendency for deliberate thinking using the Cognitive Reflection Task, Version 2 (CRT; Thomson & Oppenheimer, 2016). Participants solved four problems requiring deliberation to give the correct answer over an intuitive but wrong answer (four open‐ended questions: *If you're running a race and you pass the person in second place, what place are you in?* [intuitive answer: *first*, correct answer: *second*]; *A farmer had 15 sheep and all but 8 died. How many are left?* [intuitive answer: *seven*, correct answer: *eight*]; *Emily's father has three daughters. The first two are named April and May. What is the third daughter's name*? [intuitive answer: *June*, correct answer: *Emily*]; *How many cubic feet of dirt are there in a hole that is 3′ deep × 3′ wide × 3′ long?* [intuitive answer: *27*, correct answer: *none*]). We calculated the CRT score as one point per correct answer given: lower scores represented an increased tendency for intuitive thinking, whereas higher scores represented an increased tendency for deliberate thinking.

## RESULTS

### Statement evaluation

As expected from disinformation about climate science and discourses to delay climate policy, the 78 climate disinformation statements made participants feel negative about climate science and action (mean_affect:science_ = −0.51 ± 2.75; mean_affect:action_ = −0.57 ± 2.74, see Table 2). However, two statements made participants feel positive about climate science and actions (e.g., statement 28: *We need to have an honest conversation about balancing climate policy with energy and food security. This means connecting the question of emission reduction targets with an understanding of the technology and economic costs required to get there*.^7^).

**FIGURE 2:**
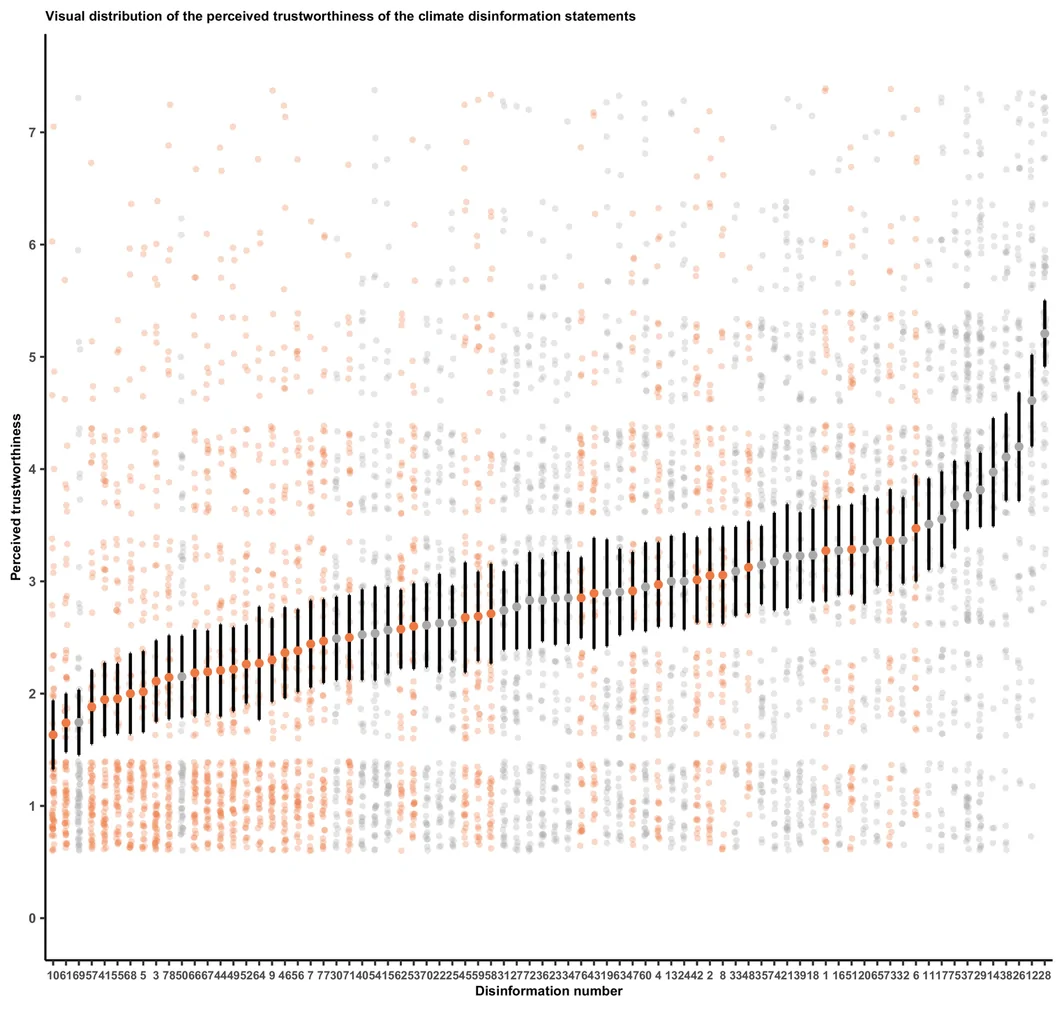
Boxplot for raw evaluations of perceived trustworthiness of each climate disinformation statement, reordered in ascending order of perceived trustworthiness for visual purposes. Discourses of delay of climate policies are represented in orange; disinformation about climate science is represented in grey. The x‐axis represents each climate disinformation statement, reordered in terms of ascending perceived persuasiveness. The y‐axis represents the ratings of how trustworthy each climate disinformation statement was perceived, with values increasing from 1 representing increasing trustworthiness. The full item list is available in Table S2.

Overall, evaluations were spread across the measurement continuum for most variables, including perceived trustworthiness ratings (see Figure 2; Figures S3–S16) and perceptions of the statements being likely to be true, with one fourth of statements believed likely to be true; these two ratings were practically indistinguishable from perceived persuasiveness (*r* = 0.94, 95% CI [0.91, 0.96]), and perceived informativeness (*r* = 0.90, 95% CI [0.85, 0.94]). Statements perceived as more trustworthy were considered less manipulative (*r* = −0.73, 95% CI [−0.82, −0.61]). Participants were more willing to share the climate disinformation statements they perceived trustworthy (*r* = 0.58, 95% CI [0.52, 0.64]); however, sharing intentions were low (mean_sharing intentions_ = 1.66 ± 1.20) for all but two statements. The statements did not generate excitement (mean_excitement_ = 1.27 ± 0.78), nor were they perceived as funny (mean_funniness_ = 1.30 ± 0.84), but evoked moderate feelings of worry (mean_worry_ = 2.85 ± 1.69) and stronger feelings of outrage (mean_outrage_ = 3.73 ± 1.55). Most climate disinformation statements were perceived as favouring conservatives (mean_slant_ = 4.15 ± 1.43), with a tenth of statements perceived as liberally slanted. Finally, as expected from decades of climate disinformation, some statements were familiar to participants, but familiarity was spread across ‘familiar’, ‘unfamiliar’, and ‘unsure’ response options. Congruently with the proposition that previously encountered disinformation is perceived as more likely to be true (Pillai & Fazio, 2021), additional multilevel analyses indicate that climate disinformation statements never encountered were perceived as less trustworthy (*F*(2, 4878.5) = 39.648, *p* < .001) than familiar climate disinformation (mean_diff_ = −0.50, *t*(4942.9) = −7.885, *β* = −0.54, 95% CI [−0.68, −0.41], *p* < .001) and statements that participants were unsure about (mean_diff_ = −0.45, *t*(4974.9) = −6.089, *β* = −0.42, 95% CI [−0.55, −0.28], *p* < .001). As expected for social media posts, the distribution of engagement metrics was skewed towards zero, with some outlier climate disinformation posts amassing thousands of likes and retweets (e.g., statement 45). Due to the small sample of climate disinformation statements, no validation measures significantly predicted the engagement metrics (Figure S1).

### Individual differences

We calculated the correlation between the within‐participant average validation scores and the four individual differences (Table 3): political ideology (economic and social), cognitive reflection (CRT‐2 scores), trust in science, and climate change beliefs (importance, worry, impacts on the self, impacts on others).

**TABLE 3:** Correlations between the individual differences and the validation measures.

	Social conservativism	Economic conservativism	CRT	Trust in science	Climate change importance	Climate change worry	Climate change impacts on self (*n* = 493)	Climate change impacts on others (*n* = 496)
Affect: climate science	−0.30*** [−0.38, −0.21]	−0.28*** [−0.36, −0.19]	0.11** [0.03, 0.20]	0.35*** [0.27, 0.42]	0.41*** [0.33, 0.48]	0.36*** [0.28, 0.43]	0.34*** [0.26, 0.41]	0.37*** [0.29, 0.44]
Affect: climate action	−0.27*** [−0.35, − 0.18]	−0.27*** [−0.35, −0.19]	0.10* [0.02, 0.19]	0.34*** [0.26, 00.42]	.42*** [0.34, 0.49]	0.37*** [0.30, 0.45]	0.29*** [0.20, 00.37]	.37*** [0.29, 0.44]
Importance	0.16*** [0.07, 0.25]	0.14** [0.05, 0.23]	−0.01 [−0.10, 0.07]	−0.06 [−0.15, 0.03]	−0.003 [−0.09, 0.08]	0.02 [−0.06, 0.11]	−0.03 [−0.11, 0.06]	0.01 [−0.08, 0.09]
Sharing intentions^†^	0.17 [0.08, 0.25]	0.12 [0.04, 0.21]	−0.15 [−0.24, −0.07]	−0.21 [−0.29, −0.13]	−0.13 [−0.21, −0.04]	−0.14 [−0.22, −0.05]	−0.05 [−0.14, 0.04]	−0.17 [−0.25, −0.08]
Perceived as being true	0.35*** [0.27, 0.42]	0.33*** [0.25, 0.41]	−0.08 [−0.17, 0.004]	−0.37*** [−0.44, −0.29]	−0.41*** [−0.48, −0.34]	−0.39*** [−0.47, −0.32]	−0.29*** [−0.37, −0.21]	−0.44*** [−0.51, −0.37]
Political slant	−0.34*** [−0.42, −0.26]	−0.27*** [0.11, 0.16]	0.13** [0.04, 0.22]	0.15*** [0.07, 0.24]	0.21*** [0.12, 0.29]	0.19*** [0.10, 0.27]	0.13** [0.04, 0.21]	0.17*** [0.08, 0.25]
Manipulative	−0.32*** [−0.39, −0.23]	−0.27*** [−0.35, −0.19]	0.10* [0.01, 0.18]	0.29*** [0.21, 0.37]	0.43*** [0.36, 0.50]	0.42*** [0.34, 0.48]	0.26*** [0.18, 0.35]	0.37*** [0.29, 0.44]
Persuasive	0.36*** [0.27, 0.43]	0.33*** [0.25, 0.41]	−0.11* [−0.19, −0.02]	−0.30*** [−0.38, −0.21]	−0.35*** [−0.42, −0.27]	−0.33*** [−0.41, −0.25]	−0.21*** [−0.30, −0.13]	−0.35*** [−0.43, −0.27]
Trustworthiness	0.36*** [0.28, 0.44]	0.33*** [0.25, 0.40]	−0.09* [−0.18, −0.01]	−0.38*** [−0.45, −0.30]	−0.40*** [−0.47, −0.33]	−0.40*** [−0.47, −0.33]	−0.27*** [−0.35, −0.19]	−0.44*** [−0.51, −0.37]
Excitement^†^	0.22 [0.13, 0.30]	0.19 [0.10, 0.28]	−0.10 [−0.19, −0.01]	−0.10 [−0.19, −0.02]	−0.15 [−0.24, −0.07]	−0.12 [−0.20, −0.03]	−0.03 [−0.12, 0.05]	−0.16 [−0.24, −0.07]
Funny^†^	0.04 [−0.05, 0.13]	0.07 [−0.02, 0.16]	−0.05 [−0.13, 0.04]	−0.06 [−0.14, −0.03]	−0.07 [−0.16, 0.02]	−0.07 [−0.16, 0.01]	0.04 [−0.04, 0.13]	−0.11 [−0.20, −0.02]
worry	−0.14** [−0.22, −0.05]	−0.14** [−0.23, −0.06]	−0.08 [−0.17, 0.003]	0.16** [0.07, 0.25]	0.31*** [0.23, 0.39]	0.33*** [0.24, 0.40]	0.29*** [0.21, 0.37]	0.24*** [0.15, 0.32]
Informative	0.31*** [0.23, 0.39]	0.28*** [0.20, 0.36]	−0.13** [−0.21, −0.04]	−0.24*** [−0.32, −0.16]	−0.26*** [−0.34, −0.17]	−0.24*** [−0.32, −0.16]	−0.12** [−0.20, −0.03]	−0.30*** [−0.38, −0.21]
Outrage	−0.19*** [−0.27, −0.10]	−0.16*** [−0.25, −0.08]	−0.02 [−0.11, 0.06]	0.18*** [0.10, 0.27]	0.32*** [0.24, 0.40]	0.36*** [0.28, 0.43]	0.21*** [0.13, 0.30]	0.25*** [0.17, 0.33]

*Note*: *p < .05. **p < .01. ***p< .001. 95% Confidence intervals in brackets. ^†^Spearman's rho.

As predicted, the more people held conservative social and economic political ideology, the more they trusted the climate disinformation statements (i.e., perceived as moderately more trustworthy, more informative, and less manipulative). Moreover, the more participants distrusted scientists, the more, on average, they trusted the climate disinformation statements, in line with previous evidence (Cologna & Siegrist, 2020; Gauchat et al., 2017; Safford et al., 2020); they furthermore felt more negatively about climate science and action. The less worried participants were of and perceived climate change as unimportant, and the more they perceived climate harms as (inter)personally distant, the more they trusted the statements and the more negative their affective reactions. Corroborating previous evidence (Pennycook et al., 2022), people predisposed towards deliberate thinking considered the climate disinformation statements less persuasive and less impactful on their affect; however, this relationship was small (Gignac & Szodorai, 2016).

**FIGURE 3:**
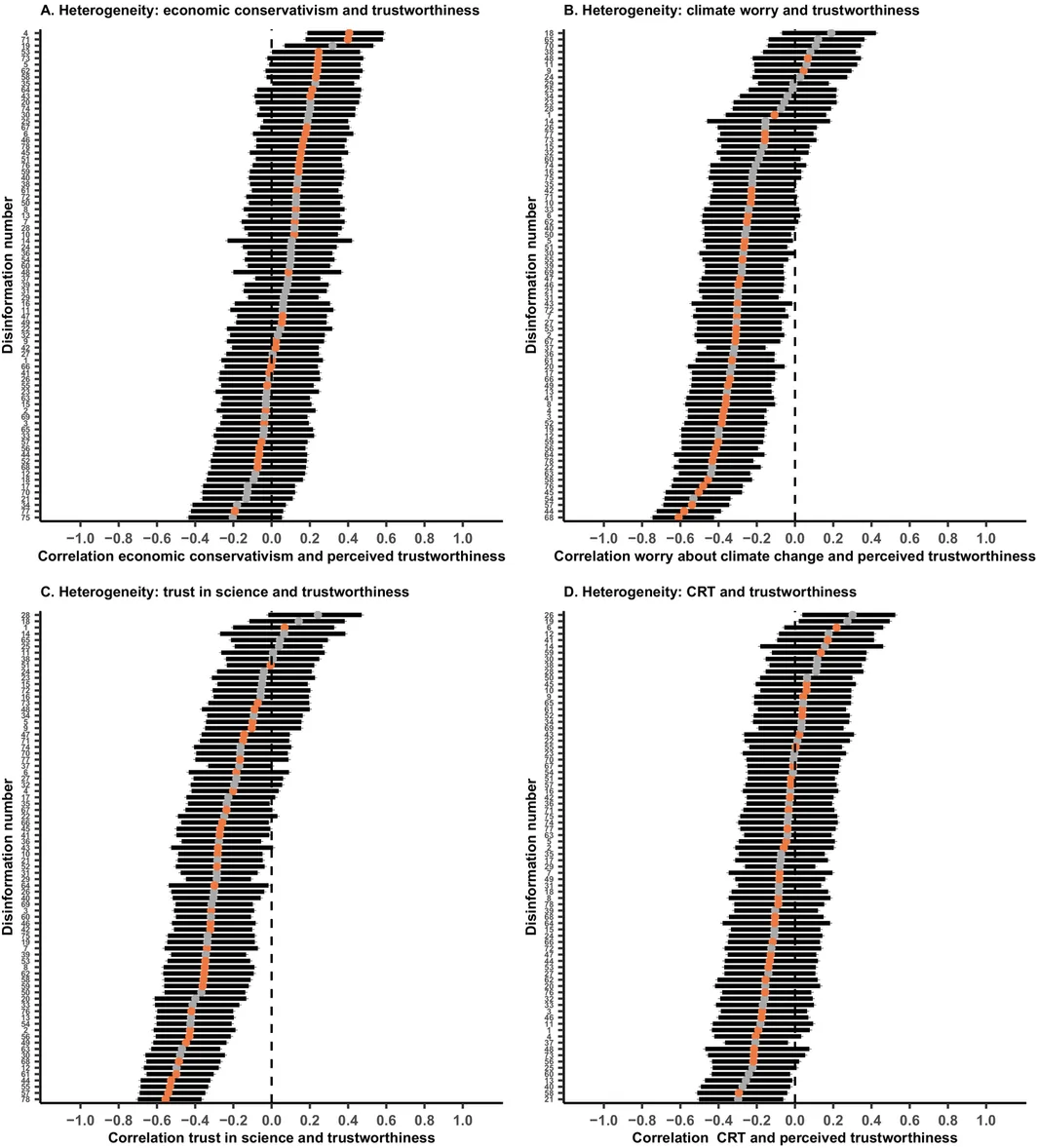
Heterogeneity of the correlation between the perceived trustworthiness of each disinformation and individual differences. Discourses of delay of climate policies are represented in orange; disinformation about climate science is represented in grey. The x‐axis represents the correlation between the perceived trustworthiness of the climate disinformation statement and the individual difference of interest. The y‐axis represents each climate disinformation statement. Horizontal bars are the 95% confidence intervals. The vertical dashed line represents *r* = 0 between perceived trustworthiness and individual differences of interest. The full item list is available in Table S2. (a) Heterogeneity of the correlation between economic conservative ideology and perceived trustworthiness. (b) Heterogeneity of the correlation between SASSY item ‘worry about climate change’ (Chryst et al., 2018) and perceived trustworthiness. (c) Heterogeneity of the correlation between trust in science and perceived trustworthiness. (d) Heterogeneity of the correlation between dispositional tendency for deliberate thinking and perceived trustworthiness.

Focusing on the rating validation measures of each climate disinformation statement, the influence of individual difference measures on the evaluation of each climate disinformation statement was very heterogeneous (Figure 3). For example, for some statements, trustworthy ratings were – descriptively – positively related to CRT performance and trust in science. In contrast, for other statements, trustworthy ratings were negatively associated with the two individual difference measures.

### Statement characteristics predicting persuasion‐related outcomes

Additional multilevel models suggested that disinformation about climate science differed significantly from discourses of delay. Accounting for the variance associated with each single climate disinformation statement and each participant as random intercepts (Judd et al., 2012) and disinformation macro‐category as a fixed factor, delay arguments were, on average, rated as more trustworthy (mean_diff_ = 0.65, *t*(77.9) = 4.918, *β* = 0.64, 95% CI [0.38, 0.90], *p* < .001), informative (mean_diff_ = 0.38, *t*(77.6) = 3.865, *β* = 0.37, 95% CI [0.18, 0.57], *p* < .001) and persuasive (mean_diff_ = 0.62, *t*(78.1) = 5.294, *β* = 0.61, 95% CI [0.39, 0.83], *p* < .001), more worrying (mean_diff_ = 0.28, *t*(75.7) = 3.890, *β* = 0.30, 95% CI [0.14, 0.45], *p* < .001), and less favourable to conservatives (mean_diff_ = −0.30, *t*(80.3) = −3.653, *β* = −0.30, 95% CI [−0.46, −0.14], *p* < .001). Participants also reported more intentions to share delay arguments over disinformation about climate science (mean_diff_ = 0.23, *t*(77.4) = 5.051, *β* = 0.23, 95% CI [0.14, 0.32], *p* < .001).

We also explored the relationship between selected evaluations of the two macro‐categories of climate disinformation and individual differences (see Tables S3 and S4). We could not detect a significant difference across the perceived importance of and the intentions to share disinformation about climate science over arguments for the delay of climate action according to individual differences (all *p*s > .001^8^). However, the more participants were worried about climate change, the less trustworthy (*t* = −3.508, *β* = −0.16, 95% CI [−0.25, −0.07], *p* < .001) and persuasive (*t* = −3.611, *β* = −0.17, 95% CI [−0.27, −0.08], *p* < .001) they found disinformation about climate science over arguments to delay climate action; moreover, the more participants worried about the effects of climate change for future generations, the less trustworthy (*t* = −4.798, *β* = −0.24, 95% CI [−0.34, −0.14], *p* < .001) and persuasive (*t* = 4.317, *β* = −0.23, 95% CI [−0.34, −0.13], *p* < .001) they found disinformation about climate science over arguments to delay climate action. Political ideology and CRT scores did not significantly moderate the evaluations of statements belonging to the different disinformation macro‐categories (all *p*s > .001).

## DISCUSSION

We conducted a study to validate the Climate Disinformation Corpus, a set of 78 climate disinformation statements to be used as stimuli for research testing climate disinformation interventions. This corpus has many advantages. First, it contains highly ecological statements used to spread climate disinformation taken from public figures of the climate change countermovement. Second, it provides a large, heterogeneous dataset of climate disinformation statements, evaluated on 15 variables and four individual difference measures of interest in disinformation research (Pennycook et al., 2021). Such a heterogeneous set can be used to overcome threats to generalizability of misinformation studies due to single, not validated stimuli (Clark, 1973; Yarkoni, 2022). Third, it contains multiple subtypes of climate disinformation and discourses of delay (Coan et al., 2021; Lamb et al., 2020). These disinformation statements can be used to mimic messages from the climate change countermovement against which multiple disinformation interventions could be tested and compared.

Across a British representative sample, the climate disinformation statements negatively affected how people feel about the science of climate change and climate action. The statements were evaluated with substantial heterogeneity across multiple influence dimensions, such as the perceived trustworthiness and persuasiveness, and the amount of outrage they evoked. Heterogeneity characterized also how individual differences influenced the evaluation of the climate disinformation statements. Whereas previous literature described the relationship between individual differences and climate belief or denial as a monolithic (e.g., Pennycook et al., 2022), we found the relationship more nuanced, with individual differences influencing the appraisal of disinformation on a case‐by‐case basis. For example, on average, trust in science was moderately and negatively correlated with the perceived trustworthiness of climate disinformation statements. Still, the relationship varied across disinformation to the extent that the directionality was reversed for some statements.

Both sources of heterogeneity stress the importance of developing and using validated sets of stimuli, such as the Climate Disinformation Corpus, for intervention research; selecting climate disinformation idiosyncratically runs the risk of using experimental stimuli that behave differently than the average climate disinformation (see Allen et al., 2024; Yarkoni, 2022). In turn, this can invalidate the study conclusions (Maertens, 2022; Maertens et al., 2024; Pennycook et al., 2021; Swire‐Thompson et al., 2022). Furthermore, these findings warrant using multiple climate disinformation stimuli within a single study to statistically account for the identified heterogeneity across climate disinformation and generalize findings outside the tested stimuli (Judd et al., 2012; Spampatti, Hahnel, et al., 2023).

### Using the climate disinformation corpus

The Climate Disinformation Corpus can be used – and the statements clustered – in multiple ways. In primis, the climate disinformation stimuli can be used as experimental materials that can mimic messages from the climate change countermovement, to then test interventions against real disinformation. For example, these stimuli can be used as disinformation messages after a psychological inoculation, to measure how the inoculation can reduce the influence of climate disinformation on climate beliefs and behaviours (Spampatti, Hahnel, et al., 2023; van der Linden et al., 2017); these stimuli can also be used as disinformation messages before debunking to measure the effectiveness of fact‐checking climate disinformation (Swire‐Thompson et al., 2022); when paired with source information, these stimuli can also be disinformation posts embedded in simulated social media environments (Jagayat & Choma, 2024), to test interventions adapted to online ecosystems (e.g., community notes, Drolsbach et al., 2024; lateral reading; Panizza et al., 2022; or changing the incentive structure of social media; Globig et al., 2023); finally, the climate disinformation can be used to measure the effects of climate disinformation messages^9^ (McCright et al., 2016; Spampatti, Hahnel, & Brosch, 2024; van der Linden et al., 2017).

Rather than excluding climate disinformation statements according to participants' evaluations, we include all pretested statements to maximize the experimental freedom of researchers wanting to conduct studies on climate disinformation. For example, we could have removed statement 26 for being the only statement whose trustworthiness was *positively* related to cognitive reflection. However, we can envision researchers wanting to test (and fight against) disinformation that selectively affects people with a high tendency for deliberation. In the following, we propose three ways the statements can be employed in research testing interventions to fight climate disinformation:

*Maximum Disinformation Per Statement*: as the name implies, this selection strategy focuses on the disinformation statements with the maximum effect on the variable of interest. For example, researchers might select those climate disinformation statements that are perceived as the most trustworthy if their aim is to sever tests interventions that increase participants' discrimination accuracy (Batailler et al., 2022), such as accuracy prompts (Pennycook & Rand, 2019).

*Mean Disinformation per Statement*: this selection strategy focuses on those statements with the average effect across one or more validation measures to test behavioural interventions against the average climate disinformation. For example, in previous work, we identified 20 climate disinformation statements that deviated the least from the average across all variables (Spampatti, Hahnel, et al., 2023). To do so, we standardized all validation measures for all climate disinformation statements. We then summed all values per each climate disinformation statement to produce a single ‘total deviance’ score per statement. We thus created a ‘mean deviance set’ of climate disinformation statements as experimental stimuli.

*Maximum variability*: as climate disinformation spans more than 50 subtypes (Coan et al., 2021; Lamb et al., 2020), some researchers may be interested in developing interventions that are generalizable across disinformation subtypes (e.g., Roozenbeek, Maertens, et al., 2022; Roozenbeek, van der Linden, et al., 2022). Thus, they may select their stimuli in a manner that varies across the largest number of disinformation subtypes.

These are three examples of implementing the Climate Disinformation Corpus in future research. The strategies can be implemented simultaneously, and we suggest that researchers who are interested in the role of individual differences select climate disinformation statements according to how their unique correlations will influence their research questions. All uses of the Climate Disinformation Corpus should contain an exhaustive debrief that explains and debunks the climate disinformation statements in detail, to avoid a continued influence of misinformation on participants beyond the laboratory settings (Ecker et al., 2022; Murphy & Greene, 2023; Swire‐Thompson et al., 2022).

### Limitations, or how to build the climate disinformation corpus 2.0

With climate disinformation evolving to dynamically supply justifications for inaction (Lamb et al., 2020; Tilsted et al., 2022), the Climate Disinformation Corpus will need to be updated. On the one hand, disinformation about climate science is decreasing in favour of discourses of delay (Coan et al., 2021). On the other hand, new climate disinformation types are entering public discourse (Lees et al., 2020), for example, against negative emission systems (Colvin et al., 2020), wildfires (Caulfield & Starbird, 2023), climate activists (Westervelt & Dembicki, 2023), or questioning the harmful environmental and health‐related effects of plastic pollution (Maquart et al., 2022; Michaels, 2020) and of extreme weather (Spring, 2024). We suggest how future validation studies of disinformation stimuli could improve our limitations.

First, we collected our climate disinformation statements from the social media platform Twitter/X. Our choice was pragmatic, for the ease and comprehensiveness of data access to public posts by elite communicators and because the argumentations behind climate disinformation spread on the platform mirror the argumentations made in other news sources (Kinol et al., 2025; Rojas et al., 2024). However, this type of data collection relies on academics being able to access social media data, access that is being increasingly revoked (Rathje, 2024; Wagner, 2023). Future data collections of disinformation statements to validate could consider scraping TikTok (Nieto‐Sandoval & Ferré‐Pavia, 2024), contrarian blogs (Coan et al., 2021), op‐eds (Elsasser & Dunlap, 2013), BlueSky (Quelle & Bovet, 2025), and other available public resources instead.

Second, our chief aim was to validate a set of climate disinformation statements to employ as experimental messages resembling the communication of climate change countermovement actors. As such, we conducted our validation study without comparing the disinformation statements with factual climate statements. Comparing the climate disinformation statements with accurate climate information can be done to measure participants' discriminant accuracy between real and false statements, which provides insights into how people process (dis)information and identify interventions improving truth discernment (Acerbi et al., 2022, Batailler et al., 2022, Pennycook & Rand, 2021; Spampatti, Hahnel, & Brosch, 2024). This issue limits the applicability of the Climate Disinformation Corpus, as it excludes using these items for the development of truth discernment tasks akin to the Misinformation Susceptibility Test (Maertens et al., 2024; see also Fischer et al., 2019; Pennycook et al., 2021). However, such a comparison can be experimentally conducted by complementing the Climate Disinformation Corpus with a set of real, accurate climate information that has also been validated. This has been recently done in an information sampling study, where 75 statements of the Climate Disinformation Corpus have been paired with accurate climate posts (Rahmani Azad et al., 2025). The authors found climate disinformation and climate information were equally persuasive in influencing participants' concern about climate change. This study provides first evidence that the validated climate disinformation statements can be used with independently collected accurate climate statements to investigate discernment.

The absence of accurate climate statements was for pragmatic reasons, as we lacked a list of climate informers akin to the list of climate change countermovement public figures and as research access to the social media platform X has been impaired (Rathje, 2024). Future studies could collect and compare evaluations across disinformation and factual climate information. The first step would be creating comprehensive lists of climate change countermovement actors and accurate climate communicators that cohabit an online social media platform. This list can be used to harvest a representative sample of their online communications (via API access or website crawlers; e.g., Bukold, 2025; Kenny, 2024). The accurate climate information statements thus collected could be validated with the same evaluation measure we employed and strengthened with more diverse individual difference measures (e.g., the aforementioned Misinformation Susceptibility Test; Maertens et al., 2024).

Third, we validated English‐based stimuli with a British sample. This choice was again pragmatic, to parsimoniously collect data from one country where we could extend their evaluations to other countries. However, disinformation is ubiquitous in climate discourse in any language (Levantesi, 2021; Mann, 2021) and this Corpus may be less appropriate for understanding the effects of climate disinformation in non‐Anglophone environments. Therefore, we welcome validations of statements in other languages, especially for information environments underrepresented in current disinformation research (Hornsey & Lewandowsky, 2022; Nguyễn et al., 2022).

Fourth, our initial computational social science data collection targeted actors known to spread disinformation about climate science and climate solutions. Although comprehensive, the denial of species extinction (Lees et al., 2020) and other topics defied categorization in existing schemas, but that will play a significant role in the future, such as grievances about loss and damage and denial of harmful effects of air and plastic pollution (Tabuchi, 2024; UNHRC, 2021), are currently underrepresented. This Corpus is therefore less appropriate to understanding these specific types of disinformation. We hope that our study will inspire broader sets of climate disinformation.

Fifth, we resorted to manual annotation to preselect the tweets containing climate disinformation and discourses of delay. Recent advances in machine learning and large language models offer some promise for automating the coding process (Rathje et al., 2024; Rojas et al., 2024). One algorithm, ChatNetZero (Hsu et al., 2024), also accurately categorized the statements from the Climate Disinformation Corpus as climate disinformation and discourses of climate delay. While their performance in recognizing subtypes of disinformation is not perfect yet (Coan et al., 2021; Rojas et al., 2024), future studies could more comprehensively map climate disinformation and have a broader selection of statements to validate.^10^

## CONCLUSION

To conclude, urgent action is needed to mitigate and adapt to the climate crisis, but decades of climate disinformation have stifled structural action. We hope the newly developed Climate Disinformation Corpus, a set of 78 validated climate disinformation statements, can aid and standardize the behavioural science effort in designing better interventions to fight climate disinformation in a warming world.

## AUTHOR CONTRIBUTIONS

**Tobia Spampatti:** Conceptualization; investigation; writing – original draft; methodology; validation; formal analysis; software; data curation; project administration; visualization. **Tobias Brosch:** Writing – review and editing; funding acquisition; methodology. **Christian Mumenthaler:** Software; investigation; data curation; writing – review and editing. **Ulf J. J. Hahnel:** Writing – review and editing; project administration; funding acquisition; methodology.

## FUNDING INFORMATION

The authors would like to extend their gratitude to the Canton of Geneva and the Service Industriels de Genève for funding this project [grant UN10765 awarded to T. B. and U. H.]. TS would like to thank the Swiss National Science Foundation for providing individual career funding (SNSF Postdoc Mobility P500PS_225577). UH would like to thank the Swiss National Science Foundation for providing individual career funding (SNSF Eccellenza PCEFPI_203283). The funders had no role in study design, data collection and analysis, decision to publish, or preparation of the manuscript.

## Supporting information


Data S1.

## Data Availability

Data, materials, and code are available at the OSF repository: https://osf.io/prxtn/?view_only=85a19bf047be4abf81beb59282d2d287. Due to the nature of the data, validation data of the Climate Disinformation Corpus^11^ is available at https://doi.org/10.23668/psycharchives.13465 (only for scientific use; https://psycharchives.org/en/about#sharing_levels).

## Footnotes

^1^
As opposed to misinformation, which is information defined in the same way as disinformation but without the intent to deceive (Wardle & Derakhshan, 2017).

^2^
See https://www.youtube.com/watch?v=7q6VLXha9OM&t=884s.

^3^
Although we focus on public figures of the climate change countermovement who have publicly voiced their opinion denying anthropogenic climate change and therefore are less at risk of posthumous identification (Hopke & Hestres, 2018), we do not provide raw Twitter data to maintain a reasonable level of anonymity. It is important to note that all statements presented in the Corpus are not meant to single out any individual or organization, but to be examples of real climate disinformation that have been spread in the past decades.

^4^
As a reminder, the online survey took place after the identification of the climate disinformation statements on Twitter (see Figure 1).

^5^
Due to a coding error, climate disinformation statements 29 and 37 were delivered twice, as were statements 66 and 54. We recoded the ratings for each statement. For those participants (*n* = 7) who were presented with duplicated items, we removed all validation ratings for the duplicated statement that was presented last, to avoid familiarity effects (Pillai & Fazio, 2021).

^6^
Most statement we coded as the misinformation super‐claim 4 “Climate solutions won't work” were also very likely of being coded as delay argumentations, due to their nature of being disinformation *that by design is being used to delay climate actions/solutions*. For the current study, we consider this type of climate disinformation as arguments to delay climate action.

^7^
To reiterate, factual statements such as this one can be considered discourses of delay when they are coopted in order intentionally and misleadingly delay necessary climate mitigation actions (Lamb et al., 2020; also found in the literature as mal‐information; Wardle & Derakhshan, 2017).

^8^
Statistical threshold corrected to better account for multiple comparisons.

^9^
Only by also including an extensive debrief (Murphy & Greene, 2023).

^10^
But will have to weigh this comprehensiveness against the rising environmental footprint of employing these methods (Li et al., 2024).
