# Supplementary material for: Blueprint of a smokescreen: Introducing the validated climate disinformation corpus for behavioural research on combating climate disinformation
Source: Br J Psychol. 2025 Jul 18;117(Suppl 1):S65–89. doi: 10.1111/bjop.70012 (PMC13543337; doi:10.1111/bjop.70012)
Supplement: Supplementary file 1 [file BJOP-117-S65-s001.docx]

**Blueprint of a smokescreen: introducing the validated Climate Disinformation Corpus for behavioral research on combating climate disinformation**

# **Supplementary Materials**

***Table SM-1 – Detailed breakdown of the disinformation about climate science and discourses of delay***

| **Disinformation Type** | **Disinformation Subtype** | ***N*** | **Description** |
| --- | --- | --- | --- |
| Climate science disinformation | Global warming/Climate change is not happening | 9 | Disinformation questioning the existence of climate change. Can contain claims such as “we are heading into an ice age”; “weather is cold”; “there is an hiatus in warming”; “Extremes aren’t increasing” |
| Climate science disinformation | Human greenhouse gas emissions are not causing global warming/climate change | 7 | Disinformation questioning the causal relationship between increasing GHGs and climate change. Can contain claims such as “it is all natural cycles” and “there is no evidence for a greenhouse effect” |
| Climate science disinformation | Climate impacts are not bad | 5 | Disinformation questioning the consequences of climate change. Can contain claims such as “CO2 is not a pollutant” and “warming of only a *few* degrees” |
| Climate science disinformation | Climate solutions won’t work | 26 | Disinformation questioning the effectiveness of climate solutions. Can contain claims such as “Policies are harmful/ineffective”; “change is too hard”; “clean energy won’t work”; “we need energy”. Category subsumed with Discourses of delay. |
| Climate science disinformation | Climate movement/science is unreliable | 25 | Disinformation questioning the reliability and objectivity of climate science/scientists and the climate movement. Can contain claims such as “Climate science/movement is unreliable” and “climate is a conspiracy” |
| Discourses of delay | Redirect Responsibility – Whataboutism | 6 | Delaying climate policy by redirecting responsibility “Our carbon footprint is trivial compared to [...]. Therefore it makes no sense for us to take action, at least until [...] does so.” |
| Discourses of delay | Push non-transformative solutions – Technological optimism | 2 | Delaying climate policy by pushing non-transformative solutions “We should focus our efforts on current and future technologies, which will unlock great possibilities for addressing climate change.” |
| Discourses of delay | Push non-transformative solutions – Fossil fuels solutionism | 5 | Delaying climate policy by pushing non-transformative solutions “Fossil fuels are part of the solution. Our fuels are becoming more efficient and are a bridge towards a low-carbon future.” |
| Discourses of delay | Push non-transformative solutions – No sticks, just carrots | 1 | Delaying climate policy by pushing non-transformative solutions “Society will only respond to supportive policies, restrictive measures will fail and should be abandoned.” |
| Discourses of delay | Emphasize the downsides – Appeal to social justice | 8 | Delaying climate policy by emphasizing the downsides “Climate actions will generate large costs. Vulnerable members of our society will be burdened; hard-working people cannot enjoy their holidays.” |
| Discourses of delay | Emphasize the downsides – Appeal to well-being | 7 | Delaying climate policy by emphasizing the downsides “Fossil fuels are required for development. Abandoning them will condemn the global poor to hardship and their right to modern livelihoods.” |
| Discourses of delay | Emphasize the downsides – Policy Perfectionism | 4 | Delaying climate policy by emphasizing the downsides “We should seek only well-crafted solutions that are supported by all affected parties; otherwise we will waste limited opportunities for adoption.” |
| Discourses of delay | Surrender – Change is impossible | 3 | Delaying climate policy by surrendering to climate change “Any measure to reduce emissions effectively would run against current ways of life or human nature and are thus impossible to implement in a democratic society.” |
| Discourses of delay | Surrender – Doomism | 4 | Delaying climate policy by surrendering to climate change “Any mitigation actions we take are too little, too late. Catastrophic climate change is already locked-in. We should adapt, or accept our fate in the hands of God or nature.” |

***Note:*** Classification systems taken from Coan et al. (2021) and Lamb et al. (2020). Descriptions in quotation marks taken from the two original papers. Sum total of disinformation does not match 78 because some climate disinformation statement fell in both types.

***Table SM-2 – List of climate disinformation statements in the Climate Disinformation Corpus, fact checked by LLM ChatNetZero (Version 1.0)***

| **Nr.** | **Statement** | **Chat NetZero Fact Check: Responses** |
| --- | --- | --- |
| 1. | Climate models predict much drying in recent decades but inconsistent with observations of greening | *The statement "Climate models predict much drying in recent decades but inconsistent with observations of greening" can be rated as 2 on the disinformation scale about climate change.* [*Corporate Climate Responsibility Monitor 2022*](https://newclimate.org/sites/default/files/2022-06/CorporateClimateResponsibilityMonitor2022.pdf#page=76)  *It contains some misleading elements as climate models do predict changes, but the inconsistency with greening observations is not universally accepted.* [*Corporate Climate Responsibility Monitor 2022*](https://newclimate.org/sites/default/files/2022-06/CorporateClimateResponsibilityMonitor2022.pdf#page=76)  *On the delay argument scale about climate change policies, it can be rated as 3.* [*Net Zero Stocktake 2022*](https://ca1-nzt.edcdn.com/Net-Zero-Tracker/Net-Zero-Stocktake-Report-2022.pdf?v=1655074300#page=9)  *The statement seems to suggest a delay in action by implying that current observations do not align with climate models, which could potentially be used as an argument to postpone climate change policies.* [*Net Zero Stocktake 2022*](https://ca1-nzt.edcdn.com/Net-Zero-Tracker/Net-Zero-Stocktake-Report-2022.pdf?v=1655074300#page=9) |
| 2. | This is not science This is theocracy To say that it is irresponsible to challenge consensus orthodoxies is to undermine science to say that science is weak and that its results have to be enforced rather than stand to criticism | *The statement "This is not science This is theocracy To say that it is irresponsible to challenge consensus orthodoxies is to undermine science to say that science is weak and that its results have to be enforced rather than stand to criticism" can be rated as 2 in terms of being disinformation about climate change.* [*Net Zero Stocktake 2022*](https://ca1-nzt.edcdn.com/Net-Zero-Tracker/Net-Zero-Stocktake-Report-2022.pdf?v=1655074300#page=9)  *It misrepresents the nature of scientific consensus, but does not directly spread false information about climate change.* [*Corporate Climate Responsibility Monitor 2022*](https://newclimate.org/sites/default/files/2022-06/CorporateClimateResponsibilityMonitor2022.pdf#page=76)  *In terms of being a delay argument about climate change policies, it can be rated as 3.* [*Net Zero Stocktake 2022*](https://ca1-nzt.edcdn.com/Net-Zero-Tracker/Net-Zero-Stocktake-Report-2022.pdf?v=1655074300#page=9)  *It suggests that current scientific consensus should be challenged, which could potentially delay the implementation of climate change policies.* [*Net Zero Stocktake 2022*](https://ca1-nzt.edcdn.com/Net-Zero-Tracker/Net-Zero-Stocktake-Report-2022.pdf?v=1655074300#page=9) |
| 3. | The past 260 billion tons of CO2 emissions have had no warming effect. | *The statement "The past 260 billion tons of CO2 emissions have had no warming effect" is rated 4 for being absolutely disinformation about climate change.* [*Corporate Climate Responsibility Monitor 2022*](https://newclimate.org/sites/default/files/2022-06/CorporateClimateResponsibilityMonitor2022.pdf#page=76)  *This is because scientific consensus and reports, including the IPCC Sixth Assessment Report, indicate that CO2 emissions contribute significantly to global warming.* [*Corporate Climate Responsibility Monitor 2023*](https://newclimate.org/sites/default/files/2023-02/NewClimate_CorporateClimateResponsibilityMonitor2023_Feb23.pdf#page=32)  *The statement can also be rated 4 for being absolutely a delay argument about climate change policies, as it undermines the urgency of reducing emissions and implementing climate change mitigation strategies.* [*Corporate Climate Responsibility Monitor 2022*](https://newclimate.org/sites/default/files/2022-06/CorporateClimateResponsibilityMonitor2022.pdf#page=97) |
| 4. | Earth has been frozen under ice and snow for 25 of its existence. During the cryogenic ice reached to the equator for some 200 million years. Volcanic upheaval eventually broke its stranglehold. This is a natural world far more chaotic than humans comprehend. CO2 played no part. | *The statement is rated 4 for being absolutely disinformation about climate change.* [*Corporate Climate Responsibility Monitor 2023*](https://newclimate.org/sites/default/files/2023-02/NewClimate_CorporateClimateResponsibilityMonitor2023_Feb23.pdf#page=60)  *It inaccurately dismisses the role of CO2 in climate change, contradicting scientific consensus that anthropogenic CO2 emissions are a primary driver of current global warming.* [*Net Zero Stocktake 2022*](https://ca1-nzt.edcdn.com/Net-Zero-Tracker/Net-Zero-Stocktake-Report-2022.pdf?v=1655074300#page=17)  *It is also rated 3 as a delay argument about climate change policies.* [*Corporate Climate Responsibility Monitor 2023*](https://newclimate.org/sites/default/files/2023-02/NewClimate_CorporateClimateResponsibilityMonitor2023_Feb23.pdf#page=60)  *While it acknowledges Earth's climatic history, it uses this to imply that human action is unnecessary or futile, potentially delaying proactive climate policies.* [*Net Zero Stocktake 2023*](https://ca1-nzt.edcdn.com/Reports/Net_Zero_Stocktake_2023.pdf?v=1689326892#page=10) |
| 5. | Nope, along with climate induced flooding, cooling, heating, hurricanes, tornados, and long periods of calm and sunny weather. They love to forget the nice weather. And then blame fires on climate. Maybe volcanoes & earthquakes too??” I guess every bad thing is caused by climate. | *The statement "Nope, along with climate induced flooding, cooling, heating, hurricanes, tornados, and long periods of calm and sunny weather. And then blame fires on climate. I guess every bad thing is caused by climate."* [*UN High‑Level Expert Group Report*](https://www.un.org/sites/un2.un.org/files/high-level_expert_group_n7b.pdf#page=11)  *is rated as 4 in terms of being related to climate change as it discusses various weather phenomena often linked to climate change.* [*Corporate Climate Responsibility Monitor 2022*](https://newclimate.org/sites/default/files/2022-06/CorporateClimateResponsibilityMonitor2022.pdf#page=97)  *However, it is also rated as 4 in terms of being a delay argument about climate change policies, as it seems to trivialize the impact of climate change and potentially delay action on climate policies.* [*Corporate Climate Responsibility Monitor 2022*](https://newclimate.org/sites/default/files/2022-06/CorporateClimateResponsibilityMonitor2022.pdf#page=97) |
| 6. | Top NASA Climate Modeler Admits Predictions Are “Mathematically Impossible” | *The statement "Top NASA Climate Modeler Admits Predictions Are “Mathematically Impossible”" is related to climate change, so it would be rated as 4 in terms of its relation to climate change.* [*Corporate Climate Responsibility Monitor 2022*](https://newclimate.org/sites/default/files/2022-06/CorporateClimateResponsibilityMonitor2022.pdf#page=79)  *However, the information provided does not give enough context to determine whether this statement is a delay argument about climate change policies.* [*Corporate Climate Responsibility Monitor 2022*](https://newclimate.org/sites/default/files/2022-06/CorporateClimateResponsibilityMonitor2022.pdf#page=86) |
| 7. | The current exceptional warming and cooling your seeing is due to the location of the Jet Stream. It's become very wavy due to the lack of Solar Energy going into the Oceans and nothing to do with Man Made CO2 | *The statement "The current exceptional warming and cooling your seeing is due to the location of the Jet Stream.* [*Net Zero Stocktake 2022*](https://ca1-nzt.edcdn.com/Net-Zero-Tracker/Net-Zero-Stocktake-Report-2022.pdf?v=1655074300#page=17)  *It's become very wavy due to the lack of Solar Energy going into the Oceans and nothing to do with Man Made CO2" is rated 4 in terms of being related to climate change as it discusses phenomena (warming, cooling, jet stream behavior) that are part of climate systems.* [*UN High‑Level Expert Group Report*](https://www.un.org/sites/un2.un.org/files/high-level_expert_group_n7b.pdf#page=6)  *However, it is also rated 4 as a delay argument about climate change policies because it dismisses the role of human-made CO2, which contradicts the consensus in climate science that human activities are a significant driver of current climate change.* [*Corporate Climate Responsibility Monitor 2022*](https://newclimate.org/sites/default/files/2022-06/CorporateClimateResponsibilityMonitor2022.pdf#page=97) |
| 8. | According to global warming theory the poles should warm significantly if carbon dioxide is driving temperatures Just the opposite is occurring in the southern hemisphere. | *The statement "According to global warming theory the poles should warm significantly if carbon dioxide is driving temperatures Just the opposite is occurring in the southern hemisphere" is rated 4 in terms of being related to climate change as it directly discusses global warming and its effects.* [*Net Zero Stocktake 2022*](https://ca1-nzt.edcdn.com/Net-Zero-Tracker/Net-Zero-Stocktake-Report-2022.pdf?v=1655074300#page=17)  *However, it is rated 2 in terms of being a delay argument about climate change policies.* [*Corporate Climate Responsibility Monitor 2022*](https://newclimate.org/sites/default/files/2022-06/CorporateClimateResponsibilityMonitor2022.pdf#page=79)  *While it questions the theory of global warming, it does not explicitly argue for delaying climate change policies.* [*UN High‑Level Expert Group Report*](https://www.un.org/sites/un2.un.org/files/high-level_expert_group_n7b.pdf#page=6) |
| 9. | Nice line from a recent Tweet Alarmists: think there is no natural climate change only AGW. So they are the real climate change deniers. But of course we know that the climate was totally static and unchanging in the 4 500 000 000 years before the Industrial Revolution. | *The statement "Nice line from a recent Tweet Alarmists: think there is no natural climate change only AGW. But of course we know that the climate was totally static and unchanging in the 4 500 000 000 years before the Industrial Revolution" is rated 4 in terms of being related to climate change as it directly discusses the topic.* [*Corporate Climate Responsibility Monitor 2022*](https://newclimate.org/sites/default/files/2022-06/CorporateClimateResponsibilityMonitor2022.pdf#page=97)  *However, it is also rated 4 as a delay argument about climate change policies, as it seems to dismiss the urgency of addressing anthropogenic global warming (AGW) by emphasizing natural climate change.* [*Corporate Climate Responsibility Monitor 2023*](https://newclimate.org/sites/default/files/2023-02/NewClimate_CorporateClimateResponsibilityMonitor2023_Feb23.pdf#page=67) |
| 10. | No sane and or honest person believes climate change or CO2 emissions are an existential threat to humanity. Not one | *The statement "No sane and or honest person believes climate change or CO2 emissions are an existential threat to humanity.*  *Not one" is rated 4 for being absolutely disinformation about climate change.* [*Corporate Climate Responsibility Monitor 2022*](https://newclimate.org/sites/default/files/2022-06/CorporateClimateResponsibilityMonitor2022.pdf#page=76)  *This is based on the consensus in the scientific community, as represented by the IPCC, that climate change is a significant threat to humanity and requires urgent action to reduce CO2 emissions.* [*Net Zero Stocktake 2023*](https://ca1-nzt.edcdn.com/Reports/Net_Zero_Stocktake_2023.pdf?v=1689326892#page=10)  *The statement is also rated 4 for being absolutely a delay argument about climate change policies, as it dismisses the need for immediate action to address climate change.* [*Corporate Climate Responsibility Monitor 2022*](https://newclimate.org/sites/default/files/2022-06/CorporateClimateResponsibilityMonitor2022.pdf#page=97) |
| 11. | Attempting to electrify everything and doing so solely with renewables will require a massive expansion of our electric grid and enough new high voltage transmission capacity to circle the Earth about 10 times | *The statement is rated 2 for being disinformation about climate change.* [*Corporate Climate Responsibility Monitor 2022*](https://newclimate.org/sites/default/files/2022-06/CorporateClimateResponsibilityMonitor2022.pdf#page=115)  *While it is true that transitioning to renewable energy will require significant expansion of the electric grid and transmission capacity, the specific claim about needing to circle the Earth 10 times seems exaggerated and lacks supporting evidence.* [*Corporate Climate Responsibility Monitor 2022*](https://newclimate.org/sites/default/files/2022-06/CorporateClimateResponsibilityMonitor2022.pdf#page=19)  *The statement is rated 3 for being a delay argument about climate change policies.* [*Corporate Climate Responsibility Monitor 2022*](https://newclimate.org/sites/default/files/2022-06/CorporateClimateResponsibilityMonitor2022.pdf#page=59)  *It implies that the transition to renewable energy is too difficult or costly, which could be used as an argument to delay action on climate change.* [*UN High‑Level Expert Group Report*](https://www.un.org/sites/un2.un.org/files/high-level_expert_group_n7b.pdf#page=30)  *However, the sources emphasize the urgency of transitioning to renewable energy and the risks of delay.* [*UN High‑Level Expert Group Report*](https://www.un.org/sites/un2.un.org/files/high-level_expert_group_n7b.pdf#page=30) |
| 12. | Celebrities claim climate change is the biggest threat to the future – but they arrive to Google's climate change summit in 114 private jets and Maserati SUVs. We guess the threat isn't that serious. | *The statement can be rated as 2 for being disinformation about climate change.* [*Corporate Climate Responsibility Monitor 2022*](https://newclimate.org/sites/default/files/2022-06/CorporateClimateResponsibilityMonitor2022.pdf#page=76)  *It uses a specific instance of perceived hypocrisy to undermine the seriousness of climate change, which is misleading.* [*Corporate Climate Responsibility Monitor 2022*](https://newclimate.org/sites/default/files/2022-06/CorporateClimateResponsibilityMonitor2022.pdf#page=4)  *However, it does not directly spread false information about climate science.* [*Corporate Climate Responsibility Monitor 2022*](https://newclimate.org/sites/default/files/2022-06/CorporateClimateResponsibilityMonitor2022.pdf#page=76)  *The statement can be rated as 3 for being a delay argument about climate change policies.* [*Net Zero Stocktake 2022*](https://ca1-nzt.edcdn.com/Net-Zero-Tracker/Net-Zero-Stocktake-Report-2022.pdf?v=1655074300#page=9)  *It uses the behavior of celebrities to suggest that climate change is not a serious issue, which could be used to justify delaying action on climate change.* [*Net Zero Stocktake 2022*](https://ca1-nzt.edcdn.com/Net-Zero-Tracker/Net-Zero-Stocktake-Report-2022.pdf?v=1655074300#page=9) |
| 13. | The Paris Climate Agreement is disastrous. It will cost at least $1 trillion per year. It will make everyone poorer – except for politicians and environmentalists. It won't, however, change the climate. | *The statement is rated 4 for being absolutely disinformation about climate change.* [*Corporate Climate Responsibility Monitor 2023*](https://newclimate.org/sites/default/files/2023-02/NewClimate_CorporateClimateResponsibilityMonitor2023_Feb23.pdf#page=57)  *The Paris Agreement is a global effort to combat climate change, and while it may have economic implications, it is not designed to make people poorer or benefit only politicians and environmentalists.* [*Corporate Climate Responsibility Monitor 2022*](https://newclimate.org/sites/default/files/2022-06/CorporateClimateResponsibilityMonitor2022.pdf#page=5)  *The claim that it won't change the climate is also false, as the agreement's goal is to limit global warming to 1.5°C.* [*Corporate Climate Responsibility Monitor 2023*](https://newclimate.org/sites/default/files/2023-02/NewClimate_CorporateClimateResponsibilityMonitor2023_Feb23.pdf#page=10)  *The statement is also rated 4 for being absolutely a delay argument about climate change policies, as it discourages action by misrepresenting the purpose and effects of the Paris Agreement.* [*Corporate Climate Responsibility Monitor 2022*](https://newclimate.org/sites/default/files/2022-06/CorporateClimateResponsibilityMonitor2022.pdf#page=5) |
| 14. | Despite Tens Of Billions Spent on Renewables And Steeper Energy Prices Europe's CO2 Output Climbs. | *The statement "Despite Tens Of Billions Spent on Renewables And Steeper Energy Prices Europe's CO2 Output Climbs" can be rated as 2 on the disinformation scale about climate change.* [*UN High‑Level Expert Group Report*](https://www.un.org/sites/un2.un.org/files/high-level_expert_group_n7b.pdf#page=11)  *It is somewhat misleading as it implies that renewable energy investments are ineffective, while ignoring other factors like fossil fuel reliance.* [*UN High‑Level Expert Group Report*](https://www.un.org/sites/un2.un.org/files/high-level_expert_group_n7b.pdf#page=11)  *However, it is based on a factual premise that CO2 emissions have risen.* [*Corporate Climate Responsibility Monitor 2022*](https://newclimate.org/sites/default/files/2022-06/CorporateClimateResponsibilityMonitor2022.pdf#page=76)  *On the delay argument scale, it can be rated as 3.* [*Corporate Climate Responsibility Monitor 2022*](https://newclimate.org/sites/default/files/2022-06/CorporateClimateResponsibilityMonitor2022.pdf#page=86)  *It suggests a negative view of renewable energy investments, which could potentially be used to argue for delaying climate change policies.* [*UN High‑Level Expert Group Report*](https://www.un.org/sites/un2.un.org/files/high-level_expert_group_n7b.pdf#page=11) |
| 15. | Imagine sacrificing 500 high-paying coal jobs, ranging up to $60,000/yr, for the climate hoax. Even if you believe in the hoax, global emissions are up 5% from pre-pandemic levels -- 90% because of China. Emissions from a single mine are insignificant. | *The statement is rated 4 for being absolutely disinformation about climate change.* [*Net Zero Tracker*](https://zerotracker.net/)  *It incorrectly labels climate change as a "hoax" and misrepresents China's role, ignoring its commitment to peak carbon dioxide emissions before 2030 and achieve net zero greenhouse gas emissions by 2060.* [*Net Zero Tracker*](https://zerotracker.net/)  *The statement is rated as 4, absolutely a delay argument about climate change policies.* [*Net Zero Tracker*](https://zerotracker.net/)  *It also inaccurately attributes 90% of global emissions increase to China, ignoring the country's efforts to peak carbon emissions before 2030 and achieve net zero greenhouse gas emissions by 2060, including all greenhouse gases, not just CO2.* [*Net Zero Tracker*](https://zerotracker.net/)  *The statement does not consider China's plans to lower carbon dioxide emissions per unit of GDP by over 65% in 2030 compared to 2005 level.* [*Net Zero Tracker*](https://zerotracker.net/) |
| 16. | Batteries are an expensive illusion. To back up a small wind farm, the batteries would cost more than 20 times than the wind farm itself... $3.6 billion for a 100MW wind farm. And then once the batteries are quickly drained, then what? | *The statement can be rated as 3 for being disinformation about climate change.* [*Corporate Climate Responsibility Monitor 2023*](https://newclimate.org/sites/default/files/2023-02/NewClimate_CorporateClimateResponsibilityMonitor2023_Feb23.pdf#page=60)  *It oversimplifies the cost and functionality of batteries in renewable energy systems, ignoring the fact that investments in battery storage systems are made to ensure self-sufficiency.* [*Corporate Climate Responsibility Monitor 2022*](https://newclimate.org/sites/default/files/2022-06/CorporateClimateResponsibilityMonitor2022.pdf#page=74)  *The statement can also be rated as 3 for being a delay argument about climate change policies.* [*Corporate Climate ResponsibilityMonitor 2023*](https://newclimate.org/sites/default/files/2023-02/NewClimate_CorporateClimateResponsibilityMonitor2023_Feb23.pdf#page=60)  *It uses the high cost of batteries as a reason to question the viability of renewable energy, which could potentially delay the transition to renewable energy systems.* [*UN High‑Level Expert Group Report*](https://www.un.org/sites/un2.un.org/files/high-level_expert_group_n7b.pdf#page=30)  *However, the text emphasizes the urgency of this transition and the need to keep the cost of capital for renewables low.* [*UN High‑Level Expert Group Report*](https://www.un.org/sites/un2.un.org/files/high-level_expert_group_n7b.pdf#page=30) |
| 17. | Green technologies like electric vehicles, wind turbines and solar panels generally require more minerals to build than their fossil fuel-based counterparts. In other words, green tech means more mining, not less. | *The statement "Green technologies like electric vehicles, wind turbines and solar panels generally require more minerals to build than their fossil fuel-based counterparts.* [*Corporate Climate Responsibility Monitor 2022*](https://newclimate.org/sites/default/files/2022-06/CorporateClimateResponsibilityMonitor2022.pdf#page=19)  *In other words, green tech means more mining, not less" can be rated as 2 on the disinformation scale.* [*Corporate Climate Responsibility Monitor 2023*](https://newclimate.org/sites/default/files/2023-02/NewClimate_CorporateClimateResponsibilityMonitor2023_Feb23.pdf#page=37)  *While it's true that green technologies require more minerals, this doesn't necessarily equate to more mining due to recycling and efficiency improvements.* [*Corporate Climate Responsibility Monitor 2022*](https://newclimate.org/sites/default/files/2022-06/CorporateClimateResponsibilityMonitor2022.pdf#page=38)  *The statement can be rated as 3 on the delay argument scale as it could be used to argue against the rapid transition to green technologies, despite the urgent need for decarbonisation to combat climate change.* [*UN High‑Level Expert Group Report*](https://www.un.org/sites/un2.un.org/files/high-level_expert_group_n7b.pdf#page=30) |
| 18. | Emissions cuts are a fool's errand. Even if Paris commitments are met 2030 emissions will be 16 higher than 2010. And no one is on track to meet Paris promises. | *The statement is rated 3 for being disinformation about climate change and 4 for being a delay argument about climate change policies.* [*Net Zero Tracker*](https://zerotracker.net/)  *The claim that "no one is on track to meet Paris promises" is misleading.* [*Net Zero Tracker*](https://zerotracker.net/)  *For instance, Paris has a detailed plan to reduce GHG emissions by 50%, reduce consumption by 35%, and reach a 45% share of renewable energy consumption by 2030.* [*Net Zero Tracker*](https://zerotracker.net/)  *Furthermore, the city aims to achieve carbon neutrality by 2050.* [*Net Zero Tracker*](https://zerotracker.net/) |
| 19. | Willfully-blind ignorance about the consequences of [the rush to green policies] – deep recessions, broken societies and millions more going hungry – doesn’t make them any less immoral. The road to hell is paved with good intentions. Bingo. | *The statement is rated 3 for being disinformation about climate change.* [*Corporate Climate Responsibility Monitor 2023*](https://newclimate.org/sites/default/files/2023-02/NewClimate_CorporateClimateResponsibilityMonitor2023_Feb23.pdf#page=8)  *It presents a skewed view of the consequences of green policies, suggesting they lead to societal harm without acknowledging their necessity for mitigating climate change.* [*UN High‑Level Expert Group Report*](https://www.un.org/sites/un2.un.org/files/high-level_expert_group_n7b.pdf#page=30)  *It also rates 4 as a delay argument about climate change policies.* [*Corporate Climate Responsibility Monitor 2022*](https://newclimate.org/sites/default/files/2022-06/CorporateClimateResponsibilityMonitor2022.pdf#page=97)  *It uses fear-based rhetoric to discourage immediate action, implying that the rush to green policies is ill-intentioned and harmful, thus advocating for a slower approach.* [*UN High‑Level Expert Group Report*](https://www.un.org/sites/un2.un.org/files/high-level_expert_group_n7b.pdf#page=30)  *This contradicts the consensus that urgent action is needed to prevent the worst impacts of climate change.* [*UN High‑Level Expert Group Report*](https://www.un.org/sites/un2.un.org/files/high-level_expert_group_n7b.pdf#page=11) |
| 20. | Fossil fuel technology is likely the greatest tech developed by man. Has improved standard of living/health more than any other tech. | *The statement "Fossil fuel technology is likely the greatest tech developed by man. Has improved standard of living/health more than any other tech" can be rated as 2 on the disinformation scale about climate change.* [*Net Zero Stocktake 2023*](https://ca1-nzt.edcdn.com/Reports/Net_Zero_Stocktake_2023.pdf?v=1689326892#page=25)  *While it's true that fossil fuel technology has significantly improved living standards, it's also clear that it's a major contributor to climate change.* [*UN High‑Level Expert Group Report*](https://www.un.org/sites/un2.un.org/files/high-level_expert_group_n7b.pdf#page=11)  *The statement can be rated as 3 on the delay argument scale about climate change policies.* [*Corporate Climate Responsibility Monitor 2022*](https://newclimate.org/sites/default/files/2022-06/CorporateClimateResponsibilityMonitor2022.pdf#page=59)  *It seems to imply that because of the benefits of fossil fuel technology, we should be slow in transitioning to cleaner energy sources, which is a delay argument.* [*UN High‑Level Expert Group Report*](https://www.un.org/sites/un2.un.org/files/high-level_expert_group_n7b.pdf#page=11) |
| 21. | Fossil fuels have helped the majority of the 8 billion people in the world to have much better lives. Over half of food production is dependent on fossil fuel derived fertilizers. The climate crazies will starve people by limiting nitrogen fertilizers. | *The statement is rated 3 for being disinformation about climate change.* [*Corporate Climate Responsibility Monitor 2022*](https://newclimate.org/sites/default/files/2022-06/CorporateClimateResponsibilityMonitor2022.pdf#page=76)  *While it's true that fossil fuels have contributed to societal development and are used in fertilizer production, the claim exaggerates the impact of reducing fossil fuel use on food production.* [*Net Zero Stocktake 2023*](https://ca1-nzt.edcdn.com/Reports/Net_Zero_Stocktake_2023.pdf?v=1689326892#page=25)  *It also ignores the urgent need for transition to renewable energy sources to mitigate climate change.* [*UN High‑Level Expert Group Report*](https://www.un.org/sites/un2.un.org/files/high-level_expert_group_n7b.pdf#page=23)  *The statement is rated 4 for being a delay argument about climate change policies.* [*Corporate Climate Responsibility Monitor 2022*](https://newclimate.org/sites/default/files/2022-06/CorporateClimateResponsibilityMonitor2022.pdf#page=97)  *It uses the importance of fossil fuels in current societal structures to argue against rapid decarbonization, which is contrary to scientific consensus on the need for immediate climate action.* [*UN High‑Level Expert Group Report*](https://www.un.org/sites/un2.un.org/files/high-level_expert_group_n7b.pdf#page=23) |
| 22. | The Net Zero agenda poses a far greater threat to our living standards than austerity 2.0. And yet so-called progressives are totally onboard with it. These people care nothing for ordinary people's lives and aspirations. | *The statement is rated 3 for being disinformation about climate change.* [*Net Zero Stocktake 2022*](https://ca1-nzt.edcdn.com/Net-Zero-Tracker/Net-Zero-Stocktake-Report-2022.pdf?v=1655074300#page=9)  *It misrepresents the Net Zero agenda as a threat to living standards, ignoring the fact that accelerated climate action offers critical benefits like community health, energy, and food security.* [*UN High‑Level Expert Group Report*](https://www.un.org/sites/un2.un.org/files/high-level_expert_group_n7b.pdf#page=11)  *It also undermines the commitments of stakeholders serious about delivering on their pledges.* [*UN High‑Level Expert Group Report*](https://www.un.org/sites/un2.un.org/files/high-level_expert_group_n7b.pdf#page=38)  *The statement is rated 4 for being a delay argument about climate change policies.* [*UN High‑Level Expert Group Report*](https://www.un.org/sites/un2.un.org/files/high-level_expert_group_n7b.pdf#page=11)  *It uses a negative portrayal of the Net Zero agenda to suggest that action should be slowed or halted, which contradicts the urgent need for near-term delivery on climate goals.* [*UN High‑Level Expert Group Report*](https://www.un.org/sites/un2.un.org/files/high-level_expert_group_n7b.pdf#page=38) |
| 23. | Renewables won't take over anytime soon. Breathless climate candaigning makes us believe world will soon run on renewables. No. Past half century renewables 13-14%. By 2050, 28% On current trends, world reaches 100 in almost two centuries | *The statement "Renewables won't take over anytime soon. Breathless climate campaigning makes us believe world will soon run on renewables. Past half century renewables 13-14%. By 2050, 28% On current trends, world reaches 100 in almost two centuries" can be rated as 2 in terms of being disinformation about climate change.* [*UN High‑Level Expert Group Report*](https://www.un.org/sites/un2.un.org/files/high-level_expert_group_n7b.pdf#page=11)  *It presents a pessimistic view of renewable energy adoption but is not entirely false.* [*UN High‑Level Expert Group Report*](https://www.un.org/sites/un2.un.org/files/high-level_expert_group_n7b.pdf#page=30)  *However, it can be rated as 3 in terms of being a delay argument about climate change policies.* [*Net Zero Stocktake 2022*](https://ca1-nzt.edcdn.com/Net-Zero-Tracker/Net-Zero-Stocktake-Report-2022.pdf?v=1655074300#page=9)  *It suggests a slow transition to renewables, which could be used to justify delaying aggressive climate policies.* [*UN High‑Level Expert Group Report*](https://www.un.org/sites/un2.un.org/files/high-level_expert_group_n7b.pdf#page=23) |
| 24. | The green agenda we're being sold is absolutely not green requiring the unprecedented mining of hitherto unimagined amounts of base and rare earth metals, also rare minerals... The never ending rape of the world we're supposed to be saving. | *The statement can be rated as 2 on the disinformation scale about climate change.* [*Corporate Climate Responsibility Monitor 2022*](https://newclimate.org/sites/default/files/2022-06/CorporateClimateResponsibilityMonitor2022.pdf#page=76)  *It acknowledges the environmental impact of mining for renewable energy resources, which is a valid concern.* [*UN High‑Level Expert Group Report*](https://www.un.org/sites/un2.un.org/files/high-level_expert_group_n7b.pdf#page=30)  *However, it oversimplifies and exaggerates the issue, ignoring the broader context of the urgent need to transition away from fossil fuels.* [*UN High‑Level Expert Group Report*](https://www.un.org/sites/un2.un.org/files/high-level_expert_group_n7b.pdf#page=11)  *The statement is a 4, absolutely a delay argument about climate change policies.* [*Net Zero Stocktake 2022*](https://ca1-nzt.edcdn.com/Net-Zero-Tracker/Net-Zero-Stocktake-Report-2022.pdf?v=1655074300#page=9)  *It criticizes the green agenda, suggesting that it leads to environmental degradation due to mining of base and rare earth metals.* [*UN High‑Level Expert Group Report*](https://www.un.org/sites/un2.un.org/files/high-level_expert_group_n7b.pdf#page=30)  *This argument implies a delay in climate action by questioning the environmental impact of renewable energy sources.* [*UN High‑Level Expert Group Report*](https://www.un.org/sites/un2.un.org/files/high-level_expert_group_n7b.pdf#page=30)  *However, the text also emphasizes the urgent need for climate action, the risks of greenwashing, and the importance of meaningful commitments to net zero targets.* [*UN High‑Level Expert Group Report*](https://www.un.org/sites/un2.un.org/files/high-level_expert_group_n7b.pdf#page=6)  *It highlights the need for rapid and deep greenhouse gas emission reductions in all sectors to limit global warming.* [*UN High‑Level Expert Group Report*](https://www.un.org/sites/un2.un.org/files/high-level_expert_group_n7b.pdf#page=19) |
| 25. | Carbon emissions from fossil fuels will hit record high in 2022. It is expensive to try to fix a 2100 problem with 2022 technology. Wouldn't try to solve today's problems with 1980s technology. Expensive to try. | *The statement "Carbon emissions from fossil fuels will hit record high in 2022. Expensive to try." can be rated as 2 in terms of being disinformation about climate change.* [*Corporate Climate Responsibility Monitor 2023*](https://newclimate.org/sites/default/files/2023-02/NewClimate_CorporateClimateResponsibilityMonitor2023_Feb23.pdf#page=32)  *It contains some factual information but also suggests that addressing climate change is too costly.* [*Corporate Climate Responsibility Monitor 2023*](https://newclimate.org/sites/default/files/2023-02/NewClimate_CorporateClimateResponsibilityMonitor2023_Feb23.pdf#page=32)  *In terms of being a delay argument about climate change policies, it can be rated as 4.* [*Corporate Climate Responsibility Monitor 2022*](https://newclimate.org/sites/default/files/2022-06/CorporateClimateResponsibilityMonitor2022.pdf#page=75)  *The statement implies that it's too early to address climate change, which contradicts scientific consensus on the urgent need for action.* [*Corporate Climate Responsibility Monitor 2023*](https://newclimate.org/sites/default/files/2023-02/NewClimate_CorporateClimateResponsibilityMonitor2023_Feb23.pdf#page=32) |
| 26. | The climate lobby has spent more than 30 years preaching apocalypse to goad countries to purge fossil fuels. But people simply aren't willing to make the sacrifices in standards of living that climate advocates insist on. | *The statement can be rated as 3 on the disinformation scale about climate change.* [*Corporate Climate Responsibility Monitor 2022*](https://newclimate.org/sites/default/files/2022-06/CorporateClimateResponsibilityMonitor2022.pdf#page=59)  *It misrepresents the urgency and scientific consensus on climate change, implying it's a scare tactic by the "climate lobby".* [*Net Zero Stocktake 2022*](https://ca1-nzt.edcdn.com/Net-Zero-Tracker/Net-Zero-Stocktake-Report-2022.pdf?v=1655074300#page=9)  *It uses the unwillingness of people to make sacrifices as a reason to delay or avoid implementing necessary climate change policies, which is a classic delay tactic.* [*Net Zero Stocktake 2022*](https://ca1-nzt.edcdn.com/Net-Zero-Tracker/Net-Zero-Stocktake-Report-2022.pdf?v=1655074300#page=9)  *The statement "The climate lobby has spent more than 30 years preaching apocalypse to goad countries to purge fossil fuels.* [*Net Zero Stocktake 2022*](https://ca1-nzt.edcdn.com/Net-Zero-Tracker/Net-Zero-Stocktake-Report-2022.pdf?v=1655074300#page=9)  *But people simply aren't willing to make the sacrifices in standards of living that climate advocates insist on" can be rated as 4 = absolutely a delay argument about climate change policies.* [*Corporate Climate Responsibility Monitor 2023*](https://newclimate.org/sites/default/files/2023-02/NewClimate_CorporateClimateResponsibilityMonitor2023_Feb23.pdf#page=10)  *The statement suggests a resistance to change and a dismissal of the urgency of climate action, which aligns with the concept of a delay argument.* [*Corporate Climate Responsibility Monitor 2023*](https://newclimate.org/sites/default/files/2023-02/NewClimate_CorporateClimateResponsibilityMonitor2023_Feb23.pdf#page=103)  *It implies that the necessary actions to combat climate change are too demanding or unrealistic, thus advocating for a delay in implementing these measures.* [*Corporate Climate Responsibility Monitor 2023*](https://newclimate.org/sites/default/files/2023-02/NewClimate_CorporateClimateResponsibilityMonitor2023_Feb23.pdf#page=10) |
| 27. | Energy literacy starts with the knowledge that renewable energy is only intermittent electricity generated from unreliable breezes and sunshine, as wind turbines and solar panels cannot manufacture anything for the 8 billion on this planet. | *The statement "Energy literacy starts with the knowledge that renewable energy is only intermittent electricity generated from unreliable breezes and sunshine, as wind turbines and solar panels cannot manufacture anything for the 8 billion on this planet" can be rated as 3 on the disinformation scale about climate change.* [*Corporate Climate Responsibility Monitor 2022*](https://newclimate.org/sites/default/files/2022-06/CorporateClimateResponsibilityMonitor2022.pdf#page=74)  *It oversimplifies the nature of renewable energy and ignores its potential and advancements.* [*Corporate Climate Responsibility Monitor 2022*](https://newclimate.org/sites/default/files/2022-06/CorporateClimateResponsibilityMonitor2022.pdf#page=74)  *It can also be rated as 3 on the delay argument scale about climate change policies, as it undermines the urgency and importance of transitioning to renewable energy sources.* [*UN High‑Level Expert Group Report*](https://www.un.org/sites/un2.un.org/files/high-level_expert_group_n7b.pdf#page=30) |
| 28. | We need to have an honest conversation about balancing climate policy with energy and food security. This means connecting the question of emission reduction targets with an understanding of the technology and economic costs required to get there. | *The statement "We need to have an honest conversation about balancing climate policy with energy and food security. This means connecting the question of emission reduction targets with an understanding of the technology and economic costs required to get there" is rated 1 for being not at all disinformation about climate change.* [*Net Zero Stocktake 2023*](https://ca1-nzt.edcdn.com/Reports/Net_Zero_Stocktake_2023.pdf?v=1689326892#page=38)  *It acknowledges the complexity of climate policy and the need for comprehensive solutions.* [*UN High‑Level Expert Group Report*](https://www.un.org/sites/un2.un.org/files/high-level_expert_group_n7b.pdf#page=11)  *However, it can be rated as 2 for being somewhat a delay argument about climate change policies.* [*Corporate Climate Responsibility Monitor 2022*](https://newclimate.org/sites/default/files/2022-06/CorporateClimateResponsibilityMonitor2022.pdf#page=75)  *While it emphasizes the need for a balanced approach, it could potentially be used to justify slower action on emission reduction targets.* [*Corporate Climate Responsibility Monitor 2022*](https://newclimate.org/sites/default/files/2022-06/CorporateClimateResponsibilityMonitor2022.pdf#page=75) |
| 29. | You can't have a sensible debate about global warming & CO2 because it's developed into a religious sect. However, one thing is certain it involves the biggest transfer of wealth from the poor to the rich. Energy costs impact the poorest most. We should focus on restoring nature | *The statement can be rated as 2 for being disinformation about climate change.* [*Corporate Climate Responsibility Monitor 2022*](https://newclimate.org/sites/default/files/2022-06/CorporateClimateResponsibilityMonitor2022.pdf#page=76)  *It can be rated as 3 for being a delay argument about climate change policies.* [*Net Zero Stocktake 2022*](https://ca1-nzt.edcdn.com/Net-Zero-Tracker/Net-Zero-Stocktake-Report-2022.pdf?v=1655074300#page=9)  *It diverts attention from the urgency of reducing emissions and transitioning to renewable energy, instead suggesting a focus on nature restoration.* [*UN High‑Level Expert Group Report*](https://www.un.org/sites/un2.un.org/files/high-level_expert_group_n7b.pdf#page=11)  *The statement ignores the fact that climate change disproportionately affects the poor and that renewable energy can provide equitable access to energy, especially in developing countries.* [*UN High‑Level Expert Group Report*](https://www.un.org/sites/un2.un.org/files/high-level_expert_group_n7b.pdf#page=30)  *It also overlooks the need for immediate action to decarbonise the entire value chain.* [*Corporate Climate Responsibility Monitor 2022*](https://newclimate.org/sites/default/files/2022-06/CorporateClimateResponsibilityMonitor2022.pdf#page=21) |
| 30. | You are lying. Fossil fuels gave us cheap energy for decades so billions live longer healthier happier lives. Many technologies like carbon capture, filters fuel additives etc reduces emissions. Banning fossil fuels is creating fuel poverty and harming people | *The statement is rated 3 for being disinformation about climate change.* [*Corporate Climate Responsibility Monitor 2022*](https://newclimate.org/sites/default/files/2022-06/CorporateClimateResponsibilityMonitor2022.pdf#page=76)  *While it's true that fossil fuels have provided cheap energy, the statement ignores the significant contribution of these fuels to global greenhouse gas emissions and climate change.* [*UN High‑Level Expert Group Report*](https://www.un.org/sites/un2.un.org/files/high-level_expert_group_n7b.pdf#page=7)  *It also overlooks the consensus from the International Energy Agency and the IPCC that deep cuts in fossil fuel consumption and production are essential for achieving net zero targets.* [*Net Zero Stocktake 2023*](https://ca1-nzt.edcdn.com/Reports/Net_Zero_Stocktake_2023.pdf?v=1689326892#page=24)  *The statement is rated 4 for being a delay argument about climate change policies.* [*Corporate Climate Responsibility Monitor 2022*](https://newclimate.org/sites/default/files/2022-06/CorporateClimateResponsibilityMonitor2022.pdf#page=97)  *It uses the potential negative impacts of transitioning away from fossil fuels as a reason to slow down or halt this necessary transition.* [*UN High‑Level Expert Group Report*](https://www.un.org/sites/un2.un.org/files/high-level_expert_group_n7b.pdf#page=23) |
| 31. | Solar and wind are far more expensive than established reliable stable secure electricity from pure hydro coal gas nuclear. That's why your shift to unreliable, unstable, expensive solar and wind; is devastating families; and exporting manufacturing jobs | *The statement is rated 4 for being absolutely disinformation about climate change.* [*UN High‑Level Expert Group Report*](https://www.un.org/sites/un2.un.org/files/high-level_expert_group_n7b.pdf#page=11)  *It inaccurately portrays solar and wind energy as unreliable and expensive, while ignoring the environmental costs of hydro, coal, gas, and nuclear energy.* [*Corporate Climate Responsibility Monitor 2022*](https://newclimate.org/sites/default/files/2022-06/CorporateClimateResponsibilityMonitor2022.pdf#page=101)  *It also fails to acknowledge the potential of renewable energy to create jobs and stimulate economic growth.* [*UN High‑Level Expert Group Report*](https://www.un.org/sites/un2.un.org/files/high-level_expert_group_n7b.pdf#page=30)  *It uses fear tactics about job loss and economic instability to discourage the transition to renewable energy, which is a critical step in mitigating climate change.* [*UN High‑Level Expert Group Report*](https://www.un.org/sites/un2.un.org/files/high-level_expert_group_n7b.pdf#page=30)  *The statement is a 4, absolutely a delay argument about climate change policies.* [*UN High‑Level Expert Group Report*](https://www.un.org/sites/un2.un.org/files/high-level_expert_group_n7b.pdf#page=30)  *It emphasizes the perceived negatives of renewable energy sources like solar and wind, such as instability and high costs, while promoting traditional energy sources like hydro, coal, gas, and nuclear as reliable and secure.* [*Corporate Climate Responsibility Monitor 2022*](https://newclimate.org/sites/default/files/2022-06/CorporateClimateResponsibilityMonitor2022.pdf#page=101)  *It suggests that transitioning to renewable energy is causing harm to families and the economy.* [*UN High‑Level Expert Group Report*](https://www.un.org/sites/un2.un.org/files/high-level_expert_group_n7b.pdf#page=30)  *This argument is used to delay the transition to renewable energy sources and slow down climate change policies.* [*UN High‑Level Expert Group Report*](https://www.un.org/sites/un2.un.org/files/high-level_expert_group_n7b.pdf#page=30) |
| 32. | Celebrities & global leaders say they care about climate change's impact on the poor. But by depriving Africa & Asia of fossil fuels, while hoarding them for themselves, global elites aren't engaged in altruism. They're inflicting green imperialism. | *The statement is a 4, absolutely a delay argument about climate change policies.* [*UN High‑Level Expert Group Report*](https://www.un.org/sites/un2.un.org/files/high-level_expert_group_n7b.pdf#page=30)  *It emphasizes the perceived negatives of renewable energy sources like solar and wind, such as instability and high costs, while promoting traditional energy sources like hydro, coal, gas, and nuclear as reliable and secure.* [*Corporate Climate Responsibility Monitor 2022*](https://newclimate.org/sites/default/files/2022-06/CorporateClimateResponsibilityMonitor2022.pdf#page=101)  *It suggests that transitioning to renewable energy is causing harm to families and the economy.* [*UN High‑Level Expert Group Report*](https://www.un.org/sites/un2.un.org/files/high-level_expert_group_n7b.pdf#page=30)  *This argument is used to delay the transition to renewable energy sources and slow down climate change policies.* [*UN High‑Level Expert Group Report*](https://www.un.org/sites/un2.un.org/files/high-level_expert_group_n7b.pdf#page=30) |
| 33. | Europe's transition to renewable energy and net zero carbon is not working, except to make life hard on average European citizens. | *The statement "Europe's transition to renewable energy and net zero carbon is not working, except to make life hard on average European citizens" can be rated as 3 on the disinformation scale about climate change and 4 on the delay argument scale about climate change policies.* [*UN High‑Level Expert Group Report*](https://www.un.org/sites/un2.un.org/files/high-level_expert_group_n7b.pdf#page=11)  *The text indicates that the European Union is actively working towards a transition to renewable energy and net zero carbon, with measures such as mandating companies to have transition plans and cracking down on greenwashing.* [*Net Zero Stocktake 2023*](https://ca1-nzt.edcdn.com/Reports/Net_Zero_Stocktake_2023.pdf?v=1689326892#page=11)  *However, the statement suggests a delay in action and undermines the progress made, hence the high rating on the delay argument scale.* [*Corporate Climate Responsibility Monitor 2022*](https://newclimate.org/sites/default/files/2022-06/CorporateClimateResponsibilityMonitor2022.pdf#page=75) |
| 34. | FACT CHECK Results of the Biden administration's extreme climate agenda cutting emissions by 44% by 2030. Annual Jobs Lost: 1.2 MILLION. Lost Economic Growth: $7.7 TRILLION. Increase in Electric Bills: 23% Increase in Gas Prices: 2$ PER YEAR | *The statement about the Biden administration's climate agenda causing job losses, economic downturn, and increased energy prices can be rated as 3 on the disinformation scale.* [*Corporate Climate Responsibility Monitor 2022*](https://newclimate.org/sites/default/files/2022-06/CorporateClimateResponsibilityMonitor2022.pdf#page=76)  *It presents a negative view of climate policies without considering their long-term benefits or the cost of inaction.* [*UN High‑Level Expert Group Report*](https://www.un.org/sites/un2.un.org/files/high-level_expert_group_n7b.pdf#page=6)  *It can also be rated as 3 on the delay argument scale, as it uses potential short-term economic impacts to argue against immediate climate action.* [*Corporate Climate Responsibility Monitor 2022*](https://newclimate.org/sites/default/files/2022-06/CorporateClimateResponsibilityMonitor2022.pdf#page=97)  *However, the UN report and IPCC findings emphasize the urgent need for significant emission reductions to limit global warming, highlighting the importance of ambitious climate policies.* [*UN High‑Level Expert Group Report*](https://www.un.org/sites/un2.un.org/files/high-level_expert_group_n7b.pdf#page=11) |
| 35. | The war on 'fossil fuels' is absurd considering the vast fields of coal/oil/gas everywhere on earth. The mantle is brimming over with it. A United Nations bid for control, cash & power has led to an energy crisis that looms as the biggest self-inflicted disaster in human history. | *The statement is rated 4 for being absolutely disinformation about climate change and 4 for being absolutely a delay argument about climate change policies.* [*Net Zero Stocktake 2022*](https://ca1-nzt.edcdn.com/Net-Zero-Tracker/Net-Zero-Stocktake-Report-2022.pdf?v=1655074300#page=11)  *The assertion that fossil fuels are abundant and the energy crisis is a self-inflicted disaster contradicts scientific consensus.* [*UN High‑Level Expert Group Report*](https://www.un.org/sites/un2.un.org/files/high-level_expert_group_n7b.pdf#page=23)  *The International Energy Agency and the IPCC emphasize the need for deep cuts in fossil fuel consumption and production to meet the Paris Agreement targets.* [*Net Zero Stocktake 2023*](https://ca1-nzt.edcdn.com/Reports/Net_Zero_Stocktake_2023.pdf?v=1689326892#page=24)  *They also highlight that the continued reliance on fossil fuels threatens economic and national security.* [*UN High‑Level Expert Group Report*](https://www.un.org/sites/un2.un.org/files/high-level_expert_group_n7b.pdf#page=23) |
| 36. | Instead of the hysterical, religious doomsday calling, this book allows us for once to take a rational approach to fossil fuels recognize their unique, irreplaceable, literally life-giving and human flourishing enabling benefits | *The statement can be rated as 3 on the scale of disinformation about climate change.* [*Corporate Climate Responsibility Monitor 2022*](https://newclimate.org/sites/default/files/2022-06/CorporateClimateResponsibilityMonitor2022.pdf#page=76)  *It downplays the urgency of climate change and the need to transition away from fossil fuels, which contradicts the consensus in climate science that fossil fuels are a major driver of climate change.* [*UN High‑Level Expert Group Report*](https://www.un.org/sites/un2.un.org/files/high-level_expert_group_n7b.pdf#page=11)  *The statement can also be rated as 4 on the scale of being a delay argument about climate change policies.* [*Corporate Climate Responsibility Monitor 2022*](https://newclimate.org/sites/default/files/2022-06/CorporateClimateResponsibilityMonitor2022.pdf#page=97)  *It suggests a slower, more cautious approach to transitioning away from fossil fuels, which could delay necessary climate action.* [*UN High‑Level Expert Group Report*](https://www.un.org/sites/un2.un.org/files/high-level_expert_group_n7b.pdf#page=11) |
| 37. | The technologies we would need to have Net Zero carbon emissions while maintaining current levels of energy use either do not exist or are not yet viable... It poses a serious threat to our living standards that no nation should be expected to tolerate. | *The statement is rated 2 for being disinformation about climate change and 3 for being a delay argument about climate change policies.* [*Net Zero Stocktake 2022*](https://ca1-nzt.edcdn.com/Net-Zero-Tracker/Net-Zero-Stocktake-Report-2022.pdf?v=1655074300#page=9)  *While it's true that some technologies for achieving net zero carbon emissions are not yet fully viable, the statement overlooks the fact that many existing strategies can significantly reduce emissions.* [*Corporate Climate Responsibility Monitor 2023*](https://newclimate.org/sites/default/files/2023-02/NewClimate_CorporateClimateResponsibilityMonitor2023_Feb23.pdf#page=53)  *It also implies that achieving net zero emissions would necessarily harm living standards, which is not supported by the text.* [*Net Zero Stocktake 2022*](https://ca1-nzt.edcdn.com/Net-Zero-Tracker/Net-Zero-Stocktake-Report-2022.pdf?v=1655074300#page=9)  *The statement could be seen as a delay argument, as it suggests that action should be postponed until more viable technologies are developed.* [*Corporate Climate Responsibility Monitor 2023*](https://newclimate.org/sites/default/files/2023-02/NewClimate_CorporateClimateResponsibilityMonitor2023_Feb23.pdf#page=53) |
| 38. | It is morally bankrupt for Europeans to expect to take Africa's fossil fuels for their own energy production but refuse to countenance African use of those same fuels for theirs | *The statement "It is morally bankrupt for Europeans to expect to take Africa's fossil fuels for their own energy production but refuse to countenance African use of those same fuels for theirs" can be rated as 2 on the disinformation scale about climate change.* [*UN High‑Level Expert Group Report*](https://www.un.org/sites/un2.un.org/files/high-level_expert_group_n7b.pdf#page=11)  *It is not entirely disinformation as it addresses the inequity in energy access between developed and developing nations.* [*UN High‑Level Expert Group Report*](https://www.un.org/sites/un2.un.org/files/high-level_expert_group_n7b.pdf#page=11)  *However, it can be rated as 3 on the delay argument scale about climate change policies.* [*Net Zero Stocktake 2022*](https://ca1-nzt.edcdn.com/Net-Zero-Tracker/Net-Zero-Stocktake-Report-2022.pdf?v=1655074300#page=9)  *It suggests a justification for continued use of fossil fuels in developing countries, which could potentially delay the global transition to renewable energy.* [*UN High‑Level Expert Group Report*](https://www.un.org/sites/un2.un.org/files/high-level_expert_group_n7b.pdf#page=23) |
| 39. | Communist China pollutes more than the Europe, US and India combined and growing. Why do they get a pass? It is very costly to end coal and replace with wind and solar that mostly is made in China. They got us coming and going. The greens are helping China. | *The statement is rated 3 for being disinformation about climate change and 3 for being a delay argument about climate change policies.* [*Net Zero Tracker*](https://zerotracker.net/)  *While it's true that China is a significant contributor to global emissions, it's misleading to suggest they are not taking action.* [*Net Zero Tracker*](https://zerotracker.net/)  *China has implemented policies to tackle environmental problems and has a net zero timeline.* [*Net Zero Tracker*](https://zerotracker.net/)  *The assertion that "greens" are helping China seems to be a delay argument, deflecting responsibility from other countries' climate actions.* [*Net Zero Tracker*](https://zerotracker.net/) |
| 40. | The dishonesty of the climate debate. Why should rich countries pay $trillions/year in climate reparations? Because "crescendo of disasters" and similar claims. Reality: fatalities and costs of climate disasters down, for rich, poor, and all countries | *The statement "The dishonesty of the climate debate Why should rich countries pay $trillions/year in climate reparations? Because "crescendo of disasters" and similar claims.  Reality: fatalities and costs of climate disasters down, for rich, poor, and all countries" can be rated as 3 in terms of being disinformation about climate change.* [*UN High‑Level Expert Group Report*](https://www.un.org/sites/un2.un.org/files/high-level_expert_group_n7b.pdf#page=11)  *It misrepresents the reality of increasing climate disasters and their costs.* [*UN High‑Level Expert Group Report*](https://www.un.org/sites/un2.un.org/files/high-level_expert_group_n7b.pdf#page=11)  *It can also be rated as 3 in terms of being a delay argument about climate change policies, as it questions the need for financial contributions from rich countries, potentially delaying necessary climate action.* [*UN High‑Level Expert Group Report*](https://www.un.org/sites/un2.un.org/files/high-level_expert_group_n7b.pdf#page=30) |
| 41. | The French Alps are snow capped in early November (just as they always have been). How can that be if CC is warming the Earth? A thinking person just might see that the whole CC thing is complete BS. Little warming with little change in climate. Relax everything is normal. | *The statement "The French Alps are snow capped in early November (just as they always have been). How can that be if CC is warming the Earth? Little warming with little change in climate.” is rated as 4 = absolutely disinformation about climate change.* [*Corporate Climate Responsibility Monitor 2022*](https://newclimate.org/sites/default/files/2022-06/CorporateClimateResponsibilityMonitor2022.pdf#page=97)  *This statement misrepresents the complex nature of climate change and its impacts.* [*UN High‑Level Expert Group Report*](https://www.un.org/sites/un2.un.org/files/high-level_expert_group_n7b.pdf#page=11)  *It also rates as 4 = absolutely a delay argument about climate change policies, as it dismisses the urgency of addressing climate change and suggests no action is needed.* [*Corporate Climate Responsibility Monitor 2022*](https://newclimate.org/sites/default/files/2022-06/CorporateClimateResponsibilityMonitor2022.pdf#page=97) |
| 42. | Latest NASA World Temperature Data for October shows the current base line 1991-2020 maintaining its confirmation that 'Global Warming' has been and gone for this Century.. it's not surprising why the out of date base between 1951-1980 is still used.. it has the highest reading. | *The statement is rated as 4 = absolutely disinformation about climate change.* [*Corporate Climate Responsibility Monitor 2022*](https://newclimate.org/sites/default/files/2022-06/CorporateClimateResponsibilityMonitor2022.pdf#page=76)  *It contradicts the consensus in climate science that global warming is ongoing and accelerating.* [*Net Zero Stocktake 2022*](https://ca1-nzt.edcdn.com/Net-Zero-Tracker/Net-Zero-Stocktake-Report-2022.pdf?v=1655074300#page=9)  *The statement is rated as 3 = somewhat a delay argument about climate change policies.* [*Corporate Climate Responsibility Monitor 2022*](https://newclimate.org/sites/default/files/2022-06/CorporateClimateResponsibilityMonitor2022.pdf#page=86)  *It implies that because global warming has supposedly ended, there is less urgency to implement climate change policies.* [*Net Zero Stocktake 2023*](https://ca1-nzt.edcdn.com/Reports/Net_Zero_Stocktake_2023.pdf?v=1689326892#page=10) |
| 43. | Today's 'global warming' is estimated to be an otherwise unmeasurable 0.4°C (0.72°F) over the 1979-2000 average... despite 50% of all manmade emissions. No 2022 weather event was unprecedented or can be blamed on CO2 emissions. | *The statement is rated as 4 = absolutely disinformation about climate change.* [*Corporate Climate Responsibility Monitor 2022*](https://newclimate.org/sites/default/files/2022-06/CorporateClimateResponsibilityMonitor2022.pdf#page=76)  *It contradicts the IPCC's findings that global warming is a significant issue and that human activities, particularly CO2 emissions, are a major contributor.* [*Corporate Climate Responsibility Monitor 2023*](https://newclimate.org/sites/default/files/2023-02/NewClimate_CorporateClimateResponsibilityMonitor2023_Feb23.pdf#page=32)  *The statement also downplays the urgency and severity of climate change impacts, which is inconsistent with the UN and IPCC reports.* [*UN High‑Level Expert Group Report*](https://www.un.org/sites/un2.un.org/files/high-level_expert_group_n7b.pdf#page=6)  *Furthermore, it is rated as 2 = somewhat a delay argument about climate change policies.* [*Corporate Climate Responsibility Monitor 2022*](https://newclimate.org/sites/default/files/2022-06/CorporateClimateResponsibilityMonitor2022.pdf#page=79)  *While it does not explicitly argue against policy action, its minimization of the problem could potentially be used to justify delaying or avoiding necessary climate action.* [*UN High‑Level Expert Group Report*](https://www.un.org/sites/un2.un.org/files/high-level_expert_group_n7b.pdf#page=11) |
| 44. | Global climate has not changed in any discernible way. At any given time at any given place, it is warmer, cooler, wetter, drier stormier, calmer than elsewhere. That is just weather. Not climate change. | *The statement "Global climate has not changed in any discernible way Not climate change.” is rated 4 for being absolutely disinformation about climate change.* [*Corporate Climate Responsibility Monitor 2022*](https://newclimate.org/sites/default/files/2022-06/CorporateClimateResponsibilityMonitor2022.pdf#page=76)  *It contradicts the scientific consensus that climate change is real and is causing extreme weather conditions.* [*UN High‑Level Expert Group Report*](https://www.un.org/sites/un2.un.org/files/high-level_expert_group_n7b.pdf#page=6)  *It is also rated 4 for being absolutely a delay argument about climate change policies, as it dismisses the urgency of addressing climate change and could potentially delay the implementation of necessary policies.* [*Corporate Climate Responsibility Monitor 2023*](https://newclimate.org/sites/default/files/2023-02/NewClimate_CorporateClimateResponsibilityMonitor2023_Feb23.pdf#page=10) |
| 45. | Earth has been warmer than today for 65% of the last 11,000 years. This scientific fact alone destroys the basis for the UN's long-running fear campaign on global warming. Cycles of warm & cold are not related to carbon dioxide levels. This hoax only exists in virtual reality. | *The statement is rated 4 for being absolutely disinformation about climate change.* [*Corporate Climate Responsibility Monitor 2022*](https://newclimate.org/sites/default/files/2022-06/CorporateClimateResponsibilityMonitor2022.pdf#page=76)  *It contradicts the scientific consensus that global warming is largely caused by human-induced increases in atmospheric CO2 levels.* [*Net Zero Stocktake 2022*](https://ca1-nzt.edcdn.com/Net-Zero-Tracker/Net-Zero-Stocktake-Report-2022.pdf?v=1655074300#page=9)  *The statement is also rated 4 for being absolutely a delay argument about climate change policies.* [*Corporate Climate Responsibility Monitor 2023*](https://newclimate.org/sites/default/files/2023-02/NewClimate_CorporateClimateResponsibilityMonitor2023_Feb23.pdf#page=10)  *It dismisses the urgency of implementing measures to reduce emissions and achieve net zero targets, which is contrary to the findings of the UN and IPCC reports that stress the need for immediate action to limit global warming to 1.5°C.* [*Corporate Climate Responsibility Monitor 2023*](https://newclimate.org/sites/default/files/2023-02/NewClimate_CorporateClimateResponsibilityMonitor2023_Feb23.pdf#page=10) |
| 46. | The big picture of the real global crisis has been missed in the madhouse of hysteria & human greed. Earth has been in transition from hothouse to icehouse for 50 million yrs. Carbon dioxide sustains life and oxygen, but it's in full retreat. Temperatures are plummeting. Science. | *The statement is rated as 4 = absolutely disinformation about climate change.* [*Corporate Climate Responsibility Monitor 2023*](https://newclimate.org/sites/default/files/2023-02/NewClimate_CorporateClimateResponsibilityMonitor2023_Feb23.pdf#page=10)  *It contradicts the scientific consensus that global temperatures are rising due to increased CO2 emissions, not plummeting.* [*UN High‑Level Expert Group Report*](https://www.un.org/sites/un2.un.org/files/high-level_expert_group_n7b.pdf#page=6)  *The statement is also rated as 3 = somewhat a delay argument about climate change policies.* [*Corporate Climate Responsibility Monitor 2023*](https://newclimate.org/sites/default/files/2023-02/NewClimate_CorporateClimateResponsibilityMonitor2023_Feb23.pdf#page=10)  *It suggests that there is no urgency to address climate change, which could be used to justify delaying action.* [*UN High‑Level Expert Group Report*](https://www.un.org/sites/un2.un.org/files/high-level_expert_group_n7b.pdf#page=11) |
| 47. | Lots of links of studies of the Medieval Warm Period that climate science deniers (alarmists) want to pretend did not exist. Because there is no explanation for natural warming during this time. Studies point out temp was warmer back then, than now. | *The statement "Lots of links of studies of the Medieval Warm Period that climate science deniers (alarmists) want to pretend did not exist. Studies point out temp was warmer back then, than now" can be rated as 3 on the scale of disinformation about climate change.* [*Net Zero Stocktake 2022*](https://ca1-nzt.edcdn.com/Net-Zero-Tracker/Net-Zero-Stocktake-Report-2022.pdf?v=1655074300#page=17)  *It contains some misleading elements, as it oversimplifies the complex nature of climate science and ignores the consensus that current warming trends are largely driven by human activities.* [*Net Zero Stocktake 2022*](https://ca1-nzt.edcdn.com/Net-Zero-Tracker/Net-Zero-Stocktake-Report-2022.pdf?v=1655074300#page=17)  *It can also be rated as 3 on the scale of being a delay argument about climate change policies, as it seems to undermine the urgency of addressing current climate change issues.* [*Corporate Climate Responsibility Monitor 2022*](https://newclimate.org/sites/default/files/2022-06/CorporateClimateResponsibilityMonitor2022.pdf#page=76) |
| 48. | New study challenges paradigm that seasonal terrestrial changes drive global carbon cycle. Sea ice dynamics correlate better they report. Science not so 'settled. | *The statement "New study challenges paradigm that seasonal terrestrial changes drive global carbon cycle. Science not so 'settled'" can be rated as 2 on the scale of disinformation about climate change.* [*Corporate Climate Responsibility Monitor 2022*](https://newclimate.org/sites/default/files/2022-06/CorporateClimateResponsibilityMonitor2022.pdf#page=76)  *It presents a different perspective on the drivers of the global carbon cycle, which is part of scientific discourse.* [*Corporate Climate Responsibility Monitor 2023*](https://newclimate.org/sites/default/files/2023-02/NewClimate_CorporateClimateResponsibilityMonitor2023_Feb23.pdf#page=60)  *However, the phrase "Science not so 'settled" could be seen as a slight attempt to undermine the consensus on climate change.* [*Corporate Climate Responsibility Monitor 2023*](https://newclimate.org/sites/default/files/2023-02/NewClimate_CorporateClimateResponsibilityMonitor2023_Feb23.pdf#page=60)  *As for being a delay argument about climate change policies, it can be rated as 1, as it doesn't directly argue against immediate policy action.* [*Corporate Climate Responsibility Monitor 2022*](https://newclimate.org/sites/default/files/2022-06/CorporateClimateResponsibilityMonitor2022.pdf#page=97) |
| 49. | NOAA cooks their temperature books. They have adjusted the data so that the present is warmer than the 1930s. Hard to sell us on CO2 being a problem when the temperature was warmer when there was a lot less CO2. If the data doesn't match the narrative, change the data. | *The statement "NOAA cooks their temperature books.* [*Corporate Climate Responsibility Monitor 2022*](https://newclimate.org/sites/default/files/2022-06/CorporateClimateResponsibilityMonitor2022.pdf#page=26)  *Hard to sell us on CO2 being a problem when the temperature was warmer when there was a lot less CO2.* [*UN High‑Level Expert Group Report*](https://www.un.org/sites/un2.un.org/files/high-level_expert_group_n7b.pdf#page=6)  *can be rated as 4 for being absolutely disinformation about climate change.* [*Corporate Climate Responsibility Monitor 2022*](https://newclimate.org/sites/default/files/2022-06/CorporateClimateResponsibilityMonitor2022.pdf#page=76)  *It can also be rated as 4 for being absolutely a delay argument about climate change policies, as it attempts to undermine the urgency of addressing CO2 emissions.* [*Corporate Climate Responsibility Monitor 2022*](https://newclimate.org/sites/default/files/2022-06/CorporateClimateResponsibilityMonitor2022.pdf#page=76) |
| 50. | Carbon dioxide emissions have no effect on world climate - even doubling to 800ppm. CO2's ability to store outgoing radiation vanishes to almost nothing after 180ppm - now 410ppm. The UN's fake climate scare campaign on CO2 emissions has caused a global energy & economic crisis. | *The statement "Carbon dioxide emissions have no effect on world climate - even doubling to 800ppm.* [*Corporate Climate Responsibility Monitor 2023*](https://newclimate.org/sites/default/files/2023-02/NewClimate_CorporateClimateResponsibilityMonitor2023_Feb23.pdf#page=32)  *CO2's ability to store outgoing radiation vanishes to almost nothing after 180ppm - now 410ppm.* [*Corporate Climate Responsibility Monitor 2022*](https://newclimate.org/sites/default/files/2022-06/CorporateClimateResponsibilityMonitor2022.pdf#page=10)  *The UN's fake climate scare campaign on CO2 emissions has caused a global energy & economic crisis" is rated 4 for being absolutely disinformation about climate change.* [*Corporate Climate Responsibility Monitor 2022*](https://newclimate.org/sites/default/files/2022-06/CorporateClimateResponsibilityMonitor2022.pdf#page=76)  *It contradicts the scientific consensus that CO2 emissions significantly contribute to global warming.* [*Corporate Climate Responsibility Monitor 2023*](https://newclimate.org/sites/default/files/2023-02/NewClimate_CorporateClimateResponsibilityMonitor2023_Feb23.pdf#page=32)  *It is also rated 4 for being absolutely a delay argument about climate change policies, as it dismisses the urgency of reducing CO2 emissions, which could delay the implementation of necessary climate policies.* [*Corporate Climate Responsibility Monitor 2022*](https://newclimate.org/sites/default/files/2022-06/CorporateClimateResponsibilityMonitor2022.pdf#page=76) |
| 51. | CO2 is major plant food. In an honest world we would be celebrating this. More food to feed 8 billion people. Add some fossil fuel derived fertilizers and harvests have increased more than 30% for nearly every food crop across the world in last 40 years. | *The statement can be rated as 3 on the disinformation scale about climate change.* [*Corporate Climate Responsibility Monitor 2023*](https://newclimate.org/sites/default/files/2023-02/NewClimate_CorporateClimateResponsibilityMonitor2023_Feb23.pdf#page=8)  *While it is true that CO2 is used by plants for photosynthesis, the oversimplification of the issue ignores the negative impacts of excessive CO2 emissions on the environment, including global warming and ocean acidification.* [*Corporate Climate Responsibility Monitor 2022*](https://newclimate.org/sites/default/files/2022-06/CorporateClimateResponsibilityMonitor2022.pdf#page=111)  *The statement can also be rated as 3 on the delay argument scale about climate change policies.* [*Corporate Climate Responsibility Monitor 2023*](https://newclimate.org/sites/default/files/2023-02/NewClimate_CorporateClimateResponsibilityMonitor2023_Feb23.pdf#page=8)  *It suggests that the increase in CO2 is beneficial and thus, indirectly argues against immediate action to reduce CO2 emissions.* [*Corporate Climate Responsibility Monitor 2023*](https://newclimate.org/sites/default/files/2023-02/NewClimate_CorporateClimateResponsibilityMonitor2023_Feb23.pdf#page=8)  *This ignores the urgent need for carbon dioxide removal efforts to achieve net-zero emissions.* [*Corporate Climate Responsibility Monitor 2023*](https://newclimate.org/sites/default/files/2023-02/NewClimate_CorporateClimateResponsibilityMonitor2023_Feb23.pdf#page=53) |
| 52. | '"Today's 'global warming' is an estimated 0.3°C (0.54°F) over the 1979-2000 average for the date... not even measurable... despite 50% of total emissions. Warm in Europe. Cool elsewhere. Just weather... and climate propaganda | *The statement "Today's 'global warming' is an estimated 0.3°C (0.54°F) over the 1979-2000 average for the date... not even measurable... despite 50% of total emissions. Just weather... and climate propaganda" is rated 4 for being absolutely disinformation about climate change.* [*Corporate Climate Responsibility Monitor 2022*](https://newclimate.org/sites/default/files/2022-06/CorporateClimateResponsibilityMonitor2022.pdf#page=97)  *It contradicts scientific consensus that global warming is a significant and measurable phenomenon.* [*Corporate Climate Responsibility Monitor 2023*](https://newclimate.org/sites/default/files/2023-02/NewClimate_CorporateClimateResponsibilityMonitor2023_Feb23.pdf#page=10)  *It also dismisses the role of human emissions in causing climate change.* [*Corporate Climate Responsibility Monitor 2023*](https://newclimate.org/sites/default/files/2023-02/NewClimate_CorporateClimateResponsibilityMonitor2023_Feb23.pdf#page=10)  *The statement is also rated 4 for being absolutely a delay argument about climate change policies.* [*Corporate Climate Responsibility Monitor 2023*](https://newclimate.org/sites/default/files/2023-02/NewClimate_CorporateClimateResponsibilityMonitor2023_Feb23.pdf#page=10)  *It undermines the urgency of climate action by attributing climate change to natural weather patterns and dismissing it as propaganda.* [*UN High‑Level Expert Group Report*](https://www.un.org/sites/un2.un.org/files/high-level_expert_group_n7b.pdf#page=6) |
| 53. | As more wind and solar are added they raise electricity prices and destabilize electric grids. Because they are part-time unreliable weather dependent sources. We want full-time electricity. Not part-time like third world countries. All for silly expensive net zero. CA pays more. | *The statement is rated 3 for being disinformation about climate change.* [*Corporate Climate Responsibility Monitor 2022*](https://newclimate.org/sites/default/files/2022-06/CorporateClimateResponsibilityMonitor2022.pdf#page=70)  *While it's true that integrating renewable energy into the grid presents challenges, it doesn't inherently raise electricity prices or destabilize grids.* [*Corporate Climate Responsibility Monitor 2023*](https://newclimate.org/sites/default/files/2023-02/NewClimate_CorporateClimateResponsibilityMonitor2023_Feb23.pdf#page=37)  *The claim that renewables are "part-time" and likening them to "third world countries" is misleading.* [*Corporate Climate Responsibility Monitor 2023*](https://newclimate.org/sites/default/files/2023-02/NewClimate_CorporateClimateResponsibilityMonitor2023_Feb23.pdf#page=109)  *The statement is rated 2 for being a delay argument about climate change policies.* [*Corporate Climate Responsibility Monitor 2022*](https://newclimate.org/sites/default/files/2022-06/CorporateClimateResponsibilityMonitor2022.pdf#page=70)  *It criticizes net zero targets and renewable energy, potentially discouraging their adoption, but doesn't explicitly argue for delaying climate action.* [*Corporate Climate Responsibility Monitor 2022*](https://newclimate.org/sites/default/files/2022-06/CorporateClimateResponsibilityMonitor2022.pdf#page=84)  *However, it does imply a preference for full-time, likely fossil fuel-based, electricity sources.* [*Corporate Climate Responsibility Monitor 2023*](https://newclimate.org/sites/default/files/2023-02/NewClimate_CorporateClimateResponsibilityMonitor2023_Feb23.pdf#page=109) |
| 54. | Net Zero makes us Net Poorer No one voted to be Net Poorer | *The statement "Net Zero makes us Net Poorer No one voted to be Net Poorer" can be rated as 2 on the disinformation scale about climate change.* [*Net Zero Stocktake 2022*](https://ca1-nzt.edcdn.com/Net-Zero-Tracker/Net-Zero-Stocktake-Report-2022.pdf?v=1655074300#page=9)  *It is not entirely disinformation as it reflects a concern about the economic implications of transitioning to net zero, but it oversimplifies the complex economic dynamics involved.* [*UN High‑Level Expert Group Report*](https://www.un.org/sites/un2.un.org/files/high-level_expert_group_n7b.pdf#page=11)  *The statement can be rated as 4 = absolutely a delay argument about climate change policies.* [*Net Zero Stocktake 2022*](https://ca1-nzt.edcdn.com/Net-Zero-Tracker/Net-Zero-Stocktake-Report-2022.pdf?v=1655074300#page=9)  *This statement suggests a reluctance to adopt or implement net zero policies due to perceived economic drawbacks, which aligns with the definition of a delay argument.* [*UN High‑Level Expert Group Report*](https://www.un.org/sites/un2.un.org/files/high-level_expert_group_n7b.pdf#page=11)  *Despite the fact that many entities have made net zero pledges, the quality and credibility of these commitments are often questionable, and many major producer countries or companies have not committed to phasing out fossil fuels.* [*Net Zero Stocktake 2023*](https://ca1-nzt.edcdn.com/Reports/Net_Zero_Stocktake_2023.pdf?v=1689326892#page=3)  *This indicates a gap between pledges and action, which can be seen as a form of delay.* [*Corporate Climate Responsibility Monitor 2023*](https://newclimate.org/sites/default/files/2023-02/NewClimate_CorporateClimateResponsibilityMonitor2023_Feb23.pdf#page=7) |
| 55. | The evidence for manmade climate change is so thin they cannot debate it. They hide behind the lie of consensus. There is no room for consensus in science. The basis is a provable hypothesis. There is not a single peer reviewed study that proves manmade CO2 is causing warming. | *The statement is rated 4 for being absolutely disinformation about climate change.* [*Corporate Climate Responsibility Monitor 2022*](https://newclimate.org/sites/default/files/2022-06/CorporateClimateResponsibilityMonitor2022.pdf#page=76)  *It contradicts the consensus in the scientific community, as represented by the Intergovernmental Panel on Climate Change (IPCC), which confirms that human activities, particularly the emission of CO2, are causing global warming.* [*Net Zero Stocktake 2022*](https://ca1-nzt.edcdn.com/Net-Zero-Tracker/Net-Zero-Stocktake-Report-2022.pdf?v=1655074300#page=9)  *The statement is also rated 2 as a delay argument about climate change policies.* [*Corporate Climate Responsibility Monitor 2022*](https://newclimate.org/sites/default/files/2022-06/CorporateClimateResponsibilityMonitor2022.pdf#page=76)  *While it does not directly argue for delaying action, its denial of the scientific consensus could be used to justify inaction or delays in implementing climate change policies.* [*UN High‑Level Expert Group Report*](https://www.un.org/sites/un2.un.org/files/high-level_expert_group_n7b.pdf#page=19) |
| 56. | As Climategate revealed and despite much pretending to the contrary: The 2°C and 1.5°C global temperature targets were 'plucked out of thin air' and are not scientific. Stalin had 5-year plans. John Kerry has Paris targets. | *The statement is rated 4 for being absolutely disinformation about climate change.* [*Net Zero Tracker*](https://zerotracker.net/)  *It attempts to discredit the Paris targets, which are based on a detailed plan to reduce GHG emissions, increase renewable energy consumption, and achieve carbon neutrality by 2050.* [*Net Zero Tracker*](https://zerotracker.net/)  *The statement is a 4, absolutely a delay argument about climate change policies.* [*Net Zero Tracker*](https://zerotracker.net/)  *It dismisses the 2°C and 1.5°C global temperature targets as unscientific and compares them to arbitrary plans, undermining the urgency and scientific basis of climate change policies.* [*Net Zero Tracker*](https://zerotracker.net/)  *This is despite the fact that Paris's target is based on a detailed plan to reduce GHG emissions by 50% and increase renewable energy consumption to 45% by 2030, with the ultimate goal of becoming a carbon-neutral city by 2050.* [*Net Zero Tracker*](https://zerotracker.net/) |
| 57. | Global Warming hits South America.. and the planet hits a Climate Emergency high of 0.3C.. wake up there is no 'Climate Emergency'.. just an insane belief that humans are evil and capitalism must be abolished and replaced with socialism to save Mother Nature.. | *The statement is rated 4 for being absolutely disinformation about climate change, as it dismisses the scientific consensus on the climate emergency and misrepresents the motivations behind climate action.* [*Net Zero Tracker*](https://zerotracker.net/)  *South Africa's commitment to a net zero target by 2050, despite financial and technological challenges, contradicts the claim that there is no 'Climate Emergency'.* [*Net Zero Tracker*](https://zerotracker.net/)  *The statement is rated as 4, indicating it is absolutely a delay argument about climate change policies.* [*Net Zero Tracker*](https://zerotracker.net/)  *This perspective undermines the urgency of addressing climate change and contradicts South Africa's commitment to moving towards a goal of net zero carbon emissions by 2050, as well as their efforts to combat climate change with support from developed countries.* [*Net Zero Tracker*](https://zerotracker.net/)  *The statement does not align with the principles of equity and common but differentiated responsibilities recognized by South Africa.* [*Net Zero Tracker*](https://zerotracker.net/) |
| 58. | Global warming experts predicted that snow would be a thing of the past by now. Yet, we keep getting healthy amounts. Plenty of places to ski. | *The statement "Global warming experts predicted that snow would be a thing of the past by now. Plenty of places to ski." can be rated as 3 on the disinformation scale about climate change.* [*Corporate Climate Responsibility Monitor 2022*](https://newclimate.org/sites/default/files/2022-06/CorporateClimateResponsibilityMonitor2022.pdf#page=97)  *It misrepresents the complexity of climate science, which does not predict uniform warming everywhere or the complete disappearance of snow.* [*Corporate Climate Responsibility Monitor 2023*](https://newclimate.org/sites/default/files/2023-02/NewClimate_CorporateClimateResponsibilityMonitor2023_Feb23.pdf#page=10)  *The statement can be rated as 4 = absolutely a delay argument about climate change policies.* [*Corporate Climate Responsibility Monitor 2023*](https://newclimate.org/sites/default/files/2023-02/NewClimate_CorporateClimateResponsibilityMonitor2023_Feb23.pdf#page=10)  *This statement misrepresents the complexity of climate change and uses anecdotal evidence to downplay the urgency of the situation, which is a common tactic in delay arguments.* [*Net Zero Stocktake 2022*](https://ca1-nzt.edcdn.com/Net-Zero-Tracker/Net-Zero-Stocktake-Report-2022.pdf?v=1655074300#page=9)  *It ignores the scientific consensus that climate change is causing more extreme and unpredictable weather patterns, including potentially heavier snowfall in some areas.* [*UN High‑Level Expert Group Report*](https://www.un.org/sites/un2.un.org/files/high-level_expert_group_n7b.pdf#page=6) |
| 59. | Too often, academic reports on climate use highly skewed data that seem to have been carefully selected to support aggressive environmental regulations. One recent and much-cited Lancet report appears deliberately deceptive. | *NA* |
| 60. | Death and privation caused by the lack of affordable energy caused by Green Energy policies will not affect the Elites at all. They want us to eat bugs, do a lot less as they carry on with their lives just as they are doing now. Climate scamsters. They should lead by example. | *The statement can be rated as 4 = absolutely disinformation about climate change, as it uses derogatory language and unfounded claims to discredit the efforts towards green energy and climate change mitigation.* [*Corporate Climate Responsibility Monitor 2023*](https://newclimate.org/sites/default/files/2023-02/NewClimate_CorporateClimateResponsibilityMonitor2023_Feb23.pdf#page=57)  *It also can be rated as 3 = somewhat a delay argument about climate change policies, as it suggests that the elites are not affected by the policies and therefore implies a need for slowing down or altering the approach to climate change policies.* [*UN High‑Level Expert Group Report*](https://www.un.org/sites/un2.un.org/files/high-level_expert_group_n7b.pdf#page=11) |
| 61. | The IPCC of the UN, the lead scamster organization admits that future climate cannot be predicted. Then goes on to predict it anyway. Think about that. They can't predict the weather a month from now. They can't predict the climate 20 years or 50 years from now! Silly really! | *The statement is rated as 4 for being absolutely disinformation about climate change.* [*Corporate Climate Responsibility Monitor 2022*](https://newclimate.org/sites/default/files/2022-06/CorporateClimateResponsibilityMonitor2022.pdf#page=76)  *The IPCC, a scientific body under the UN, does make predictions about future climate based on scientific models and data.* [*Net Zero Stocktake 2023*](https://ca1-nzt.edcdn.com/Reports/Net_Zero_Stocktake_2023.pdf?v=1689326892#page=3)  *It does not claim that future climate cannot be predicted.* [*Corporate Climate Responsibility Monitor 2023*](https://newclimate.org/sites/default/files/2023-02/NewClimate_CorporateClimateResponsibilityMonitor2023_Feb23.pdf#page=57)  *The statement is also rated as 4 for being absolutely a delay argument about climate change policies.* [*Corporate Climate Responsibility Monitor 2022*](https://newclimate.org/sites/default/files/2022-06/CorporateClimateResponsibilityMonitor2022.pdf#page=97)  *It attempts to undermine the credibility of the IPCC and the urgency of addressing climate change, which could delay the implementation of necessary policies.* [*UN High‑Level Expert Group Report*](https://www.un.org/sites/un2.un.org/files/high-level_expert_group_n7b.pdf#page=19) |
| 62. | February 2014: NYTimes runs an op-ed titled ""The End of Snow?" November 2022: Demand for ski homes strongest in 8 years. No climate apocalyptic has ever predicted anything correctly. | *The statement "February 2014: NYTimes runs an op-ed titled ""The End of Snow?" November 2022: Demand for ski homes strongest in 8 years. No climate apocalyptic has ever predicted anything correctly."* [*Corporate Climate Responsibility Monitor 2023*](https://newclimate.org/sites/default/files/2023-02/NewClimate_CorporateClimateResponsibilityMonitor2023_Feb23.pdf#page=57)  *can be rated as 3 on the disinformation scale about climate change.* [*Corporate Climate Responsibility Monitor 2022*](https://newclimate.org/sites/default/files/2022-06/CorporateClimateResponsibilityMonitor2022.pdf#page=76)  *However, it can be rated as 2 on the delay argument scale about climate change policies.* [*Corporate Climate Responsibility Monitor 2022*](https://newclimate.org/sites/default/files/2022-06/CorporateClimateResponsibilityMonitor2022.pdf#page=79)  *It does not directly argue against the need for climate policies but could be used to undermine the urgency of climate action by suggesting predictions are always incorrect.* [*Corporate Climate Responsibility Monitor 2023*](https://newclimate.org/sites/default/files/2023-02/NewClimate_CorporateClimateResponsibilityMonitor2023_Feb23.pdf#page=57) |
| 63. | The "Climate Crisis"(TM) is being used as an excuse to grab more control over everyone's lives. You won't own a petrol car, you won't eat red meat and your bank will track your carbon footprint, but you can bet the richest and most powerful will still be eating T-Bones. | *The statement "The "Climate Crisis"(TM) is being used as an excuse to grab more control over everyone's lives. You won't own a petrol car, you won't eat red meat and your bank will track your carbon footprint, but you can bet the richest and most powerful will still be eating T-Bones" can be rated as 4 = absolutely disinformation about climate change.* [*Corporate Climate Responsibility Monitor 2023*](https://newclimate.org/sites/default/files/2023-02/NewClimate_CorporateClimateResponsibilityMonitor2023_Feb23.pdf#page=57)  *It misrepresents the urgency and necessity of climate action, suggesting it's a control mechanism rather than a response to a real and pressing issue.* [*Net Zero Stocktake 2022*](https://ca1-nzt.edcdn.com/Net-Zero-Tracker/Net-Zero-Stocktake-Report-2022.pdf?v=1655074300#page=9)  *It can also be rated as 3 on the delay argument scale, as it implies that climate policies should be resisted or slowed down due to perceived unfairness.* [*UN High‑Level Expert Group Report*](https://www.un.org/sites/un2.un.org/files/high-level_expert_group_n7b.pdf#page=11) |
| 64. | We can all see climate is not changing or warming. We don't need lectures in a long-running fear campaign from the United Nations, a front for socialism & globalism. This politically charged scam is a bid to reset the world economy by punishing, blaming & degrading the west. | *The statement is rated 4 for being absolutely disinformation about climate change, as it contradicts the scientific consensus that climate change is real, human-induced, and a serious threat.* [*Corporate Climate Responsibility Monitor 2022*](https://newclimate.org/sites/default/files/2022-06/CorporateClimateResponsibilityMonitor2022.pdf#page=76)  *It also dismisses the role of the United Nations, which is a key actor in coordinating global climate action.* [*UN High‑Level Expert Group Report*](https://www.un.org/sites/un2.un.org/files/high-level_expert_group_n7b.pdf#page=19)  *The statement is also rated 4 for being absolutely a delay argument about climate change policies.* [*Corporate Climate Responsibility Monitor 2022*](https://newclimate.org/sites/default/files/2022-06/CorporateClimateResponsibilityMonitor2022.pdf#page=97)  *It uses politically charged language to discredit climate science and policy, suggesting that action on climate change is unnecessary and even harmful, which could delay the urgent action needed to mitigate climate change.* [*UN High‑Level Expert Group Report*](https://www.un.org/sites/un2.un.org/files/high-level_expert_group_n7b.pdf#page=19) |
| 65. | At Climate Summit, Elites Chow Down on Gourmet Meats While Telling Us to Eat Bugs | *The statement "At Climate Summit, Elites Chow Down on Gourmet Meats While Telling Us to Eat Bugs" can be rated as 2 on the disinformation scale about climate change.* [*UN High‑Level Expert Group Report*](https://www.un.org/sites/un2.un.org/files/high-level_expert_group_n7b.pdf#page=6)  *It is somewhat misleading as it oversimplifies the complex issue of dietary changes needed for climate mitigation.* [*Corporate Climate Responsibility Monitor 2023*](https://newclimate.org/sites/default/files/2023-02/NewClimate_CorporateClimateResponsibilityMonitor2023_Feb23.pdf#page=8)  *On the delay argument scale, it can be rated as 3.* [*Corporate Climate Responsibility Monitor 2022*](https://newclimate.org/sites/default/files/2022-06/CorporateClimateResponsibilityMonitor2022.pdf#page=86)  *It implies a criticism of climate policies without offering constructive solutions, which can be seen as a tactic to delay action on climate change.* [*Net Zero Stocktake 2022*](https://ca1-nzt.edcdn.com/Net-Zero-Tracker/Net-Zero-Stocktake-Report-2022.pdf?v=1655074300#page=9) |
| 66. | The UN's climate hoax has been rewarded with global media fanfare & our weak & clueless leaders. It's a crushing attack on our hard won freedom, democracy & rights. Millions of our bravest died in 2 world wars to stop such an iron fist over our lives. It's here once more. | *The statement is rated 4 for being absolutely disinformation about climate change.* [*Corporate Climate Responsibility Monitor 2022*](https://newclimate.org/sites/default/files/2022-06/CorporateClimateResponsibilityMonitor2022.pdf#page=76)  *It falsely labels the UN's climate change efforts as a hoax and undermines the scientific consensus on the issue.* [*Corporate Climate Responsibility Monitor 2022*](https://newclimate.org/sites/default/files/2022-06/CorporateClimateResponsibilityMonitor2022.pdf#page=10)  *It also scores 4 for being absolutely a delay argument about climate change policies.* [*Corporate Climate Responsibility Monitor 2022*](https://newclimate.org/sites/default/files/2022-06/CorporateClimateResponsibilityMonitor2022.pdf#page=97)  *It uses emotionally charged language and historical references to distract from the urgency of addressing climate change, suggesting that action should be delayed to protect other values.* [*UN High‑Level Expert Group Report*](https://www.un.org/sites/un2.un.org/files/high-level_expert_group_n7b.pdf#page=11)  *This contradicts the scientific consensus that immediate action is needed to limit global warming to 1.5°C and achieve net zero emissions by mid-century.* [*UN High‑Level Expert Group Report*](https://www.un.org/sites/un2.un.org/files/high-level_expert_group_n7b.pdf#page=11) |
| 67. | The climate hoax devised by the UN, supported by rich elitists is endorsed by our treacherous leaders is an attack on freedoms & rights. Climate cultism is a form of global self hatred. It aims to punish western nations by transferring huge reparations to the developing world. | *The statement "The climate hoax devised by the UN, supported by rich elitists is endorsed by our treacherous leaders is an attack on freedoms & rights.* [*UN High‑Level Expert Group Report*](https://www.un.org/sites/un2.un.org/files/high-level_expert_group_n7b.pdf#page=6)  *It aims to punish western nations by transferring huge reparations to the developing world" is rated 4 for being absolutely disinformation about climate change.* [*Corporate Climate Responsibility Monitor 2023*](https://newclimate.org/sites/default/files/2023-02/NewClimate_CorporateClimateResponsibilityMonitor2023_Feb23.pdf#page=8)  *It denies the scientific consensus on climate change and misrepresents the intentions and actions of those working to mitigate it.* [*Corporate Climate Responsibility Monitor 2022*](https://newclimate.org/sites/default/files/2022-06/CorporateClimateResponsibilityMonitor2022.pdf#page=10)  *The statement is also rated 4 for being absolutely a delay argument about climate change policies, as it seeks to undermine and delay action on climate change by spreading misinformation and fostering distrust.* [*Net Zero Stocktake 2022*](https://ca1-nzt.edcdn.com/Net-Zero-Tracker/Net-Zero-Stocktake-Report-2022.pdf?v=1655074300#page=9) |
| 68. | This is a portrait of climate fraud, posturing as the saviours of the world. They are a breed of crooks, getting rich by ripping off gullible western nations. The UN led climate hoax has been running since 1988. They want us to believe a pack of lies about earth's climate. | *The statement is rated 4 for being absolutely disinformation about climate change, as it falsely labels climate change as a hoax and undermines the scientific consensus on the issue.* [*Corporate Climate Responsibility Monitor 2022*](https://newclimate.org/sites/default/files/2022-06/CorporateClimateResponsibilityMonitor2022.pdf#page=76)  *It is also rated 4 for being absolutely a delay argument about climate change policies, as it attempts to discredit the efforts of organizations and governments to address climate change, thereby potentially delaying the implementation of necessary policies.* [*Net Zero Stocktake 2022*](https://ca1-nzt.edcdn.com/Net-Zero-Tracker/Net-Zero-Stocktake-Report-2022.pdf?v=1655074300#page=9) |
| 69. | The Bible calls for a principle of stewardship where God's people are advised to use the resources of the earth for the betterment of people's livelihood. Among those resources are fossil fuel reserves. | *The statement is rated 2 for being disinformation about climate change and 4 for being a delay argument about climate change policies.* [*Corporate Climate Responsibility Monitor 2022*](https://newclimate.org/sites/default/files/2022-06/CorporateClimateResponsibilityMonitor2022.pdf#page=76)  *While the Bible may call for stewardship, the argument misrepresents the role of fossil fuels in climate change.* [*UN High‑Level Expert Group Report*](https://www.un.org/sites/un2.un.org/files/high-level_expert_group_n7b.pdf#page=7)  *The International Energy Agency and the IPCC have clearly stated that fossil fuel consumption and production must be significantly reduced to achieve net zero emissions and limit global warming.* [*Net Zero Stocktake 2023*](https://ca1-nzt.edcdn.com/Reports/Net_Zero_Stocktake_2023.pdf?v=1689326892#page=24)  *Furthermore, the argument is a delay tactic as it suggests continued use of fossil fuels, which contradicts the urgent need for transition to renewable energy sources.* [*UN High‑Level Expert Group Report*](https://www.un.org/sites/un2.un.org/files/high-level_expert_group_n7b.pdf#page=11) |
| 70. | No one intends to cut emissions. That's why emissions targets are set for the year 2050. 2050 is a ruse for leaders to dodge having to make suicidal cuts. They also hope that Greta the Climate Puppet will have gotten a life by then. | *The statement is rated 3 for being disinformation about climate change and 4 for being a delay argument about climate change policies.* [*Corporate Climate Responsibility Monitor 2023*](https://newclimate.org/sites/default/files/2023-02/NewClimate_CorporateClimateResponsibilityMonitor2023_Feb23.pdf#page=32)  *The claim that "no one intends to cut emissions" is misleading as many entities have set net zero targets.* [*Corporate Climate Responsibility Monitor 2023*](https://newclimate.org/sites/default/files/2023-02/NewClimate_CorporateClimateResponsibilityMonitor2023_Feb23.pdf#page=32)  *However, the criticism about the 2050 target is somewhat valid as some companies and governments are indeed falling short of their interim 2030 targets and may be delaying meaningful action.* [*Corporate Climate Responsibility Monitor 2022*](https://newclimate.org/sites/default/files/2022-06/CorporateClimateResponsibilityMonitor2022.pdf#page=75)  *The derogatory reference to Greta Thunberg as a "Climate Puppet" is a clear attempt to discredit climate activism and delay policy action.* [*Corporate Climate Responsibility Monitor 2023*](https://newclimate.org/sites/default/files/2023-02/NewClimate_CorporateClimateResponsibilityMonitor2023_Feb23.pdf#page=32) |
| 71. | Radical environmentalists want to make energy more expensive so their preferred and less reliable sources of electricity can finally compete in the marketplace. They want to pick the 'energy winners' and in the process make us all 'energy losers'. | *The statement is rated 4 for being absolutely disinformation about climate change.* [*UN High‑Level Expert Group Report*](https://www.un.org/sites/un2.un.org/files/high-level_expert_group_n7b.pdf#page=6)  *It falsely portrays environmentalists as wanting to make energy more expensive and less reliable, which contradicts the text's emphasis on the need for affordable, renewable energy.* [*UN High‑Level Expert Group Report*](https://www.un.org/sites/un2.un.org/files/high-level_expert_group_n7b.pdf#page=30)  *The statement is also rated 4 for being absolutely a delay argument about climate change policies.* [*UN High‑Level Expert Group Report*](https://www.un.org/sites/un2.un.org/files/high-level_expert_group_n7b.pdf#page=11)  *It suggests slowing down the transition to renewable energy, which contradicts the text's urgency for immediate action to decarbonise the global economy and the need to accelerate the energy transition to a renewable future.* [*UN High‑Level Expert Group Report*](https://www.un.org/sites/un2.un.org/files/high-level_expert_group_n7b.pdf#page=23) |
| 72. | Greta Thunberg says she's anti-capitalist because she cares about people and the environment but that's absurd free markets have lifted millions out of poverty and reduced emissions. The real reason elites hate capitalism is because it threatens their power. | *The statement is a 3 on the disinformation scale about climate change.* [*Corporate Climate Responsibility Monitor 2022*](https://newclimate.org/sites/default/files/2022-06/CorporateClimateResponsibilityMonitor2022.pdf#page=76)  *It misrepresents Greta Thunberg's stance and oversimplifies the relationship between capitalism, poverty, and emissions.* [*UN High‑Level Expert Group Report*](https://www.un.org/sites/un2.un.org/files/high-level_expert_group_n7b.pdf#page=19)  *It's also a 3 on the delay argument scale about climate change policies.* [*Net Zero Stocktake 2022*](https://ca1-nzt.edcdn.com/Net-Zero-Tracker/Net-Zero-Stocktake-Report-2022.pdf?v=1655074300#page=9)  *It deflects from the urgency of climate action by framing it as a threat to capitalism and power structures, rather than addressing the environmental and social implications of climate change.* [*UN High‑Level Expert Group Report*](https://www.un.org/sites/un2.un.org/files/high-level_expert_group_n7b.pdf#page=11) |
| 73. | Truth is the First Casualty of Global Warming. The truth about climate change is nuanced: it is real, and in the long term it will be a problem, but its impact is less than we might believe. | *The statement "Truth is the First Casualty of Global Warming. The truth about climate change is nuanced: it is real, and in the long term it will be a problem, but its impact is less than we might believe" can be rated as 2 on the disinformation scale about climate change.* [*UN High‑Level Expert Group Report*](https://www.un.org/sites/un2.un.org/files/high-level_expert_group_n7b.pdf#page=6)  *It acknowledges the reality of climate change but downplays its impact, which contradicts the provided information about the urgent and severe consequences of climate change.* [*UN High‑Level Expert Group Report*](https://www.un.org/sites/un2.un.org/files/high-level_expert_group_n7b.pdf#page=6)  *The statement suggests that the problem is not immediate, which could be used to justify delaying climate change policies.* [*UN High‑Level Expert Group Report*](https://www.un.org/sites/un2.un.org/files/high-level_expert_group_n7b.pdf#page=11)  *The statement can be rated as 4 = absolutely a delay argument about climate change policies.* [*UN High‑Level Expert Group Report*](https://www.un.org/sites/un2.un.org/files/high-level_expert_group_n7b.pdf#page=6)  *The statement undermines the urgency of the climate crisis, contradicts scientific consensus, and could potentially delay necessary action.* [*Corporate Climate Responsibility Monitor 2023*](https://newclimate.org/sites/default/files/2023-02/NewClimate_CorporateClimateResponsibilityMonitor2023_Feb23.pdf#page=10)  *The provided text emphasizes the immediate and severe impacts of climate change, the need for rapid emission reductions, and the current shortfall in meeting these targets.* [*UN High‑Level Expert Group Report*](https://www.un.org/sites/un2.un.org/files/high-level_expert_group_n7b.pdf#page=11) |
| 74. | Wind failure driving up natural gas and electricity prices in Europe. It's expensive to rely on wind... and it's even more expensive when wind fails. The problem hasn't even started yet. Europe will face a very tight winter. | *The statement "Wind failure driving up natural gas and electricity prices in Europe.* [*Corporate Climate Responsibility Monitor 2022*](https://newclimate.org/sites/default/files/2022-06/CorporateClimateResponsibilityMonitor2022.pdf#page=73)  *It's expensive to rely on wind... and it's even more expensive when wind fails.* [*UN High‑Level Expert Group Report*](https://www.un.org/sites/un2.un.org/files/high-level_expert_group_n7b.pdf#page=30)  *can be rated as 2 in terms of being disinformation about climate change.* [*Corporate Climate Responsibility Monitor 2022*](https://newclimate.org/sites/default/files/2022-06/CorporateClimateResponsibilityMonitor2022.pdf#page=70)  *It contains some elements of truth, as renewable energy sources can be intermittent, but it oversimplifies the complexity of energy markets and the role of renewables.* [*Corporate Climate Responsibility Monitor 2022*](https://newclimate.org/sites/default/files/2022-06/CorporateClimateResponsibilityMonitor2022.pdf#page=73)  *In terms of being a delay argument about climate change policies, it can be rated as 3.* [*Corporate Climate Responsibility Monitor 2022*](https://newclimate.org/sites/default/files/2022-06/CorporateClimateResponsibilityMonitor2022.pdf#page=70)  *It suggests a negative view of renewable energy and implies a need to rely on fossil fuels, which could delay the transition to cleaner energy sources.* [*UN High‑Level Expert Group Report*](https://www.un.org/sites/un2.un.org/files/high-level_expert_group_n7b.pdf#page=23) |
| 75. | House hearing on climate treaty: Eliminating US CO2 emissions might reduce global temp by only 0.1C by 2100. | *The statement "House hearing on climate treaty: Eliminating US CO2 emissions might reduce global temp by only 0.1C by 2100" is rated 2 for disinformation about climate change.* [*Net Zero Tracker*](https://zerotracker.net/)  *It's partially misleading as it downplays the impact of US emissions reduction, but it's not entirely false.* [*Net Zero Tracker*](https://zerotracker.net/)  *It suggests that US actions might not have a significant impact, which could be used to argue against urgent emission reduction efforts.* [*Net Zero Tracker*](https://zerotracker.net/)  *However, the US has set targets to reduce emissions by 52% by 2030 and achieve net-zero emissions by 2050.*  *The statement can be rated as 4 = absolutely a delay argument about climate change policies.* [*Net Zero Tracker*](https://zerotracker.net/)  *This statement seems to downplay the impact of US emissions reduction on global temperature, potentially discouraging aggressive climate change policies.* [*Net Zero Tracker*](https://zerotracker.net/)  *Despite the seemingly small temperature reduction, the US has set a target of a net-zero emissions economy by 2050 and an interim target of reducing emissions by 52.0% relative to 2005 emissions by 2030.* [*Net Zero Tracker*](https://zerotracker.net/)  *These targets are in line with global efforts to mitigate climate change.* |
| 76. | 2016: Already Almost 50 New Peer-Reviewed Papers Refuting Alarmist CO2 Science. Show Natural Cycles Indisputable | *The statement "2016: Already Almost 50 New Peer-Reviewed Papers Refuting Alarmist CO2 Science. Show Natural Cycles Indisputable" can be rated as 4 = absolutely disinformation about climate change.* [*Corporate Climate Responsibility Monitor 2022*](https://newclimate.org/sites/default/files/2022-06/CorporateClimateResponsibilityMonitor2022.pdf#page=76)  *This is because the overwhelming consensus in the scientific community, as evidenced by the Intergovernmental Panel on Climate Change's (IPCC) reports, is that human-induced CO2 emissions are a major driver of climate change.* [*Net Zero Stocktake 2023*](https://ca1-nzt.edcdn.com/Reports/Net_Zero_Stocktake_2023.pdf?v=1689326892#page=10)  *The statement can also be rated as 3 on the scale of being a delay argument about climate change policies, as it seems to undermine the urgency of addressing CO2 emissions.* [*Corporate Climate Responsibility Monitor 2022*](https://newclimate.org/sites/default/files/2022-06/CorporateClimateResponsibilityMonitor2022.pdf#page=76) |
| 77. | Earth's temperature have been made with flawed and oversimplified internal physical assumptions. | *The statement "Earth's temperature have been made with flawed and oversimplified internal physical assumptions" can be rated as 3 on the scale of disinformation about climate change.* [*Corporate Climate Responsibility Monitor 2022*](https://newclimate.org/sites/default/files/2022-06/CorporateClimateResponsibilityMonitor2022.pdf#page=26)  *It implies a significant misunderstanding or misrepresentation of the complex scientific processes involved in climate modeling.* [*Corporate Climate Responsibility Monitor 2022*](https://newclimate.org/sites/default/files/2022-06/CorporateClimateResponsibilityMonitor2022.pdf#page=26)  *However, it doesn't completely deny the existence of climate change.* [*UN High‑Level Expert Group Report*](https://www.un.org/sites/un2.un.org/files/high-level_expert_group_n7b.pdf#page=6)  *On the delay argument scale, it can be rated as 2.* [*Corporate Climate Responsibility Monitor 2022*](https://newclimate.org/sites/default/files/2022-06/CorporateClimateResponsibilityMonitor2022.pdf#page=26)  *While it doesn't directly argue against immediate climate action, it could be used to undermine the urgency of addressing climate change by suggesting that our understanding of the problem is flawed.* [*UN High‑Level Expert Group Report*](https://www.un.org/sites/un2.un.org/files/high-level_expert_group_n7b.pdf#page=6) |
| 78. | 97% of climate models say that 97% of climate scientists are wrong | *The statement "97% of climate models say that 97% of climate scientists are wrong" is rated as 4 = absolutely disinformation about climate change.* [*Corporate Climate Responsibility Monitor 2022*](https://newclimate.org/sites/default/files/2022-06/CorporateClimateResponsibilityMonitor2022.pdf#page=76)  *This statement contradicts the consensus within the scientific community that human-induced climate change is real.* [*Net Zero Stocktake 2022*](https://ca1-nzt.edcdn.com/Net-Zero-Tracker/Net-Zero-Stocktake-Report-2022.pdf?v=1655074300#page=9)  *It also rates as 4 = absolutely a delay argument about climate change policies, as it undermines the urgency and importance of implementing climate change policies by suggesting that the majority of climate scientists are incorrect in their findings.* [*Net Zero Stocktake 2022*](https://ca1-nzt.edcdn.com/Net-Zero-Tracker/Net-Zero-Stocktake-Report-2022.pdf?v=1655074300#page=9) |

# **SM 1 – Procedure for the AI-assisted validation of the 78 climate disinformation statements**

As most of the research using large language models has focused on generating responses to climate disinformation (e.g., Zanartu et al., 2024), detecting climate disinformation remains largely untested. It is therefore possible that ChatNetZero only provides alarmed responses to prompts asking to evaluate the accuracy of input statements. We tested this concern by asking ChatNetZero to rate 30 randomly sampled statements from the Climate Information Corpus (Rahmani Azad et al., in preparation). Akin to the Climate Disinformation Corpus, the Climate Information Corpus is a validated dataset of verifiably accurate statements about climate change and climate policies that have been collected ecologically from posts on Twitter by previously identified accurate climate communicators. In short, is the epistemic opposite of the dataset of focus in the current article. We therefore tested the reliability of ChatNetZero in accurately identifying climate disinformation and its false positive rates.

ChatNetZero did not consider any of the 30 statements from the Climate Information Corpus as climate disinformation. This strongly suggests the algorithm is not simply operating by being alarmed of all incoming information as climate disinformation. Furthermore, ChatNetZero considered 21 of the statements as not being arguments to delay climate action; 5 items were not rated, as they were purely scientific arguments that did not contain any information about climate mitigation (e.g., statement 3 *Human influence on the climate system is clear. IPCC WG1.*). Four items were considered as possibly being arguments of delay. However, this rating was due to the fact that most statements did not provide more context about the climate mitigation actions they depicted. For example, statement 128 (*Forests are our life support system. Yet, they are being destroyed to make way for food commodities like palm oil, soy meat and dairy products. To win the fight against the climate crisis, we must protect forests around the world. Learn more*) was considered as somewhat a delay argument because “While it correctly identifies the need for forest protection, it does not address the necessity of reducing emissions from other sectors, potentially delaying comprehensive climate change policies”. This suggests that ChatNetZero is moderately accurate in detecting what is not arguments of delay.

Overall, ChatNetZero is broadly accurate in detecting climate disinformation statements while relying more on contextual information to decide whether a statement is a delay statement or not.

# **Supplementary Images**

***SM 2 - Correlation between the fifteen validation measures***

**Figure SM-1: Heatmap of the correlations between the average rating per each climate disinformation statement across the validation measures and the engagement metrics on social media Twitter.** From left to right (bottom to top): number of likes on Twitter/X; number of retweets on Twitter/X; affect towards climate science; affect towards climate action; perceived importance, intentions to share; perceived likelihood of the climate disinformation statement being true; perceived political favorability to conservatives; perceived manipulativeness; perceived persuasiveness, perceived trustworthiness; induced excitement; perceived funniness; induced worry; perceived informativeness; evoked outrage.

**Figure SM-2: Heatmap of the correlations between all participants’ ratings.** From left to right (bottom to top): affect towards climate science; affect towards climate action; perceived importance, intentions to share; perceived likelihood of the climate disinformation statement being true; perceived political favorability to conservatives; perceived manipulativeness; perceived persuasiveness, perceived trustworthiness; induced excitement; perceived funniness; induced worry; perceived informativeness; evoked outrage.

***SM 3 - Visual representation of the distribution of ratings across all validation measures***

**Figure SM-3: Boxplot for raw affective reactions towards climate action generated by each climate disinformation statement, reordered in descending order of affect towards climate mitigation action for visual purposes.** Discourses of delay of climate policies are represented in orange; disinformation about climate science is represented in grey . The x-axis represents each climate disinformation statement, reordered in terms of descending affect towards climate mitigation action. The y-axis represents the ratings of how people felt towards climate mitigation action after the climate disinformation statement, with values <6 representing feeling negative about climate mitigation actions, and values >5 representing feeling positive about climate mitigation actions.

**Figure SM-4: Boxplot for raw affective reactions towards climate science generated by each climate disinformation statement, reordered in descending order of affect towards climate science for visual purposes.** Discourses of delay of climate policies are represented in orange; disinformation about climate science is represented in grey. The x-axis represents each climate disinformation statement, reordered in terms of descending affect towards climate science. The y-axis represents the ratings of how people felt towards climate science after the climate disinformation statement, with values <6 representing feeling negative about climate science, and values >5 representing feeling positive about climate science.

**Figure SM-5: Boxplot for raw perceived importance of each climate disinformation statement, reordered in ascending order of perceived importance for visual purposes.** Discourses of delay of climate policies are represented in orange; disinformation about climate science is represented in grey. The x-axis represents each climate disinformation statement, reordered in terms of ascending perceived importance. The y-axis represents the ratings of perceived importance of the climate disinformation statement, with values <4 representing perceiving the climate disinformation statement as increasingly unimportant, and values >3 representing perceiving the climate disinformation statement as increasingly important.

**Figure SM-6: Boxplot for sharing intentions per each climate disinformation statement, reordered in ascending order of intentions to share for visual purposes.** Discourses of delay of climate policies are represented in orange; disinformation about climate science is represented in grey. The x-axis represents each climate disinformation statement, reordered in terms of ascending intentions to share the climate disinformation statement. The y-axis represents the intentions to share the climate disinformation statement, with values <3 representing sharing the climate disinformation statement as increasingly unlikely, and values >4 representing sharing the climate disinformation statement as increasingly likely.

**Figure SM-7: Boxplot for raw evaluations of perception of each climate disinformation statement being true, reordered in ascending order for visual purposes.** Discourses of delay of climate policies are represented in orange; disinformation about climate science is represented in grey. The x-axis represents each climate disinformation statement, reordered in terms of ascending perceived likelihood of the statement being true. The y-axis represents the perceived likelihood of the statement being true, with values <4 representing the climate disinformation statement as increasingly unlikely, and values >3 representing the climate disinformation statement as being perceived as increasingly likely.

**Figure SM-8: Boxplot for raw evaluations of the perceived political slant of each climate disinformation statement, reordered in an ascending order from favorability for liberals to favorability for conservatives for visual purposes.** Discourses of delay of climate policies are represented in orange; disinformation about climate science is represented in grey. The x-axis represents each climate disinformation statement, reordered in ascending perceived favorability from liberals to conservatives. The y-axis represents the perceived political slant, with values <4 representing the climate disinformation statement as being perceived as increasingly favorable toward liberals, and values >3 representing the climate disinformation statement as increasingly favorable toward conservatives.

**Figure SM-9: Boxplot for raw evaluations of perceived manipulativeness of each climate disinformation statement, reordered in an ascending perceived manipulativeness for visual purposes.** Discourses of delay of climate policies are represented in orange; disinformation about climate science is represented in grey. The x-axis represents each climate disinformation statement, reordered in terms of ascending perceived manipulativeness. The y-axis represents the perceived manipulativeness, with increasing values representing increasing perceived manipulativeness.

**Figure SM-10: Boxplot for raw evaluations of perceived persuasiveness of each climate disinformation statement, reordered in an ascending perceived persuasiveness for visual purposes.** Discourses of delay of climate policies are represented in orange; disinformation about climate science is represented in grey. The x-axis represents each climate disinformation statement, reordered in terms of ascending perceived persuasiveness. The y-axis represents perceived persuasiveness, with increasing values representing increasing perceived persuasiveness.

**Figure SM-11: Boxplot for raw evaluations of perceived familiarity of each climate disinformation statement, reordered in an ascending perceived familiarity for visual purposes.** Discourses of delay of climate policies are represented in orange; disinformation about climate science is represented in grey. The x-axis represents each climate disinformation statement, reordered in terms of ascending perceived familiarity. The y-axis represents the perceived familiarity, with -1 representing statements participants were *not* familiar with, 0 representing statements participants were *unsure* whether they were familiar with or not, and 1 representing statements participants were familiar with.

**Figure SM-12: Boxplot for raw feelings of excitement evoked by each climate disinformation statement, reordered in an ascending evoked excitement for visual purposes.** Discourses of delay of climate policies are represented in orange; disinformation about climate science is represented in grey. The x-axis represents each climate disinformation statement, reordered in terms of ascending excitement. The y-axis represents the evoked excitement, with increasing values representing increasing excitement.

**Figure SM-13: Boxplot for raw evaluations of how fun each climate disinformation statement was perceived to be, reordered in ascending order of perceived funniness for visual purposes.** Discourses of delay of climate policies are represented in orange; disinformation about climate science is represented in grey. The x-axis represents each climate disinformation statement, reordered in terms of ascending perceived funniness. The y-axis represents how funny each climate disinformation statement was perceived, with increasing values representing climate disinformation statements being perceived as increasingly funny.

**Figure SM-14: Boxplot for raw feelings of worry evoked by each climate disinformation statement, reordered in an ascending evoked worry for visual purposes.** Discourses of delay of climate policies are represented in orange; disinformation about climate science is represented in grey. The x-axis represents each climate disinformation statement, reordered in terms of ascending feelings of worry. The y-axis represents the evoked worry, with increasing values representing increasing worry.

**Figure SM-15: Boxplot for raw evaluations of perceived informativeness of each climate disinformation statement, reordered in ascending perceived informativeness for visual purposes.** Discourses of delay of climate policies are represented in orange; disinformation about climate science is represented in grey. The x-axis represents each climate disinformation statement, reordered in terms of ascending perceived informativeness. The y-axis represents the perceived informativeness, with increasing values representing increasing perceived informativeness.

**Figure SM-16: Boxplot for raw feelings of outrage evoked by each climate disinformation statement, reordered in an ascending evoked worry for visual purposes.** Discourses of delay of climate policies are represented in orange; disinformation about climate science is represented in grey. The x-axis represents each climate disinformation statement, reordered in terms of ascending feelings of outrage. The y-axis represents the evoked outrage, with increasing values representing increasing outrage.

**Figure SM-17: Violin plot for responses to the four items of the Six Americas Super Short Survey.** The x-axis represents each item; From left to right: perceived harm of climate change to future generations (1 = *not at all* to 4 = *a great deal*); perceived harm of climate change to the self (1 = *not at all* to 4 = *a great deal*); perceived importance of climate change (1 = *not at all important* to 5 = *extremely important*); worry about climate change (1 = *not at all worried* to 4 = *very worried*). The y-axis represents participants’ response options.

# **Supplementary Analyses – Moderation**

**Table SM-3 – Predicting perceived trustworthiness by the moderation between worry about climate change to future generations and disinformation macro-categories.**

| ***Perceived trustworthiness*** | | | | | | | ***Perceived persuasiveness*** | | | | | |
| --- | --- | --- | --- | --- | --- | --- | --- | --- | --- | --- | --- | --- |
|  |  |  |  | 95% Confidence Intervals | |  |  |  |  | 95% Confidence Intervals | |  |
| Predictor | Estimate | SE | t-value | Lower | Upper | *p* | Estimate | SE | t-value | Lower | Upper | *p* |
| Intercept | 4.48 | 0.20 | 22.551 | 4.09 | 4.87 | <.001 | 4.52 | 0.22 | 20.581 | 4.09 | 4.95 | <.001 |
| Macro-category: Science | -0.14 | 0.19 | -0.737 | -0.52 | 0.24 | .46 | -0.06 | 0.19 | -0.330 | -0.44 | 0.31 | .74 |
| SASSY worry | -0.42 | 0.06 | -7.666 | -0.53 | -0.31 | <.001 | -0.38 | 0.06 | -6.044 | -0.51 | -0.26 | <.001 |
| Macro-category: Science * SASSY worry | -0.16 | 0.05 | -3.508 | -0.25 | -0.07 | <.001 | -0.17 | 0.05 | 3.611 | -0.27 | -0.08 | <.001 |

**Table SM-4 – Predicting perceived trustworthiness by the moderation between perceived harm of climate change to future generations and disinformation macro-categories.**

| ***Perceived trustworthiness*** | | | | | | | ***Perceived persuasiveness*** | | | | | |
| --- | --- | --- | --- | --- | --- | --- | --- | --- | --- | --- | --- | --- |
|  |  |  |  | 95% Confidence Intervals | |  |  |  |  | 95% Confidence Intervals | |  |
| Predictor | Estimate | SE | t-value | Lower | Upper | *p* | Estimate | SE | t-value | Lower | Upper | *p* |
| Intercept | 4.98 | 0.24 | 21.186 | 4.52 | 5.45 | <.001 | 4.90 | 0.27 | 18.367 | 4.38 | 5.43 | <.001 |
| Macro-category: Science | 0.22 | 0.22 | 0.981 | -0.22 | 0.65 | .33 | 0.22 | 0.22 | 0.961 | -0.22 | 0.65 | .34 |
| SASSY others | -0.51 | 0.06 | -8.567 | -0.63 | -0.40 | <.001 | -0.45 | 0.07 | -6.368 | -0.58 | -0.31 | <.001 |
| Macro-category: Science * SASSY others | -0.24 | 0.05 | -4.798 | -0.34 | -0.14 | <.001 | -0.23 | 0.05 | -4.317 | -0.34 | -0.13 | <.001 |

# **Debriefing**

*Thank you very much for participating in this study!*

*The aim of this study was to understand how misinformation about climate change/global warming spread on social media. That’s right,* ***all social media posts you evaluated were misinformation about climate change/global warming!***

*Please remember that, regardless of the posts you have read, over* [***99% of climate scientists agree that climate change is real***](https://iopscience.iop.org/article/10.1088/1748-9326/ac2966) ***and it is humanly caused*** *by greenhouse gas emissions due to the burning of fossil fuels****.*** *The* [*IPCC report*](https://www.ipcc.ch/report/sixth-assessment-report-working-group-i/)*, the most authoritative body of climate change evidence, concluded that “****It is unequivocal that human influence has warmed the atmosphere, ocean and land****. Widespread and rapid changes in the atmosphere, ocean, cryosphere and biosphere have occurred.”*

*If you encounter statements that doubt the existence of human-caused climate change in the future,* ***remember that climate science is now settled, and that climate change is real and humanly caused.***

*With this study, we hope to develop ways to fight misinformation about climate change spread by vested interests.*

*If you want to write a comment about this study, you can do so here (if you want your data to be removed, please comment with “I wish for my participation to be removed from the current study, and I do not consent to the use of my data anymore.”):*

*Thank you for your participation, please click the arrow to end the experiment and be redirected to the panel provider’s website.*

# **Questionnaire**

Consent

**INFORMATION TO THE PARTICIPANTS AND CONSENT**

 **GENERAL OBJECTIVES & REQUIREMENTS**
 This is a study about climate messages. We can only give you all the details at the end of this experiment, but we will ask you to evaluate different statements about climate change/global warming that can be encountered on social media. This study aims to create and validate a set of climate messages that can be used in future studies to understand how to better communicate about climate change.

 Please be aware that some items of this survey will touch on your own political beliefs. There are no right or wrong answers. We simply ask you to respond sincerely. If you feel uncomfortable disclosing your personal beliefs, or wish to interrupt the survey for other reasons, you can stop the questionnaire at any time by pressing the “Quit Survey” button on the bottom of each page. There will be no consequences if you quit the survey.

 **PROCEDURE AND TIME INVOLVEMENT**
 The questionnaire should take you approximately 10 minutes. Before you continue to the next pages, please make sure you allocate the required amount of time in a quiet area to be able to finish them all. If you choose not to participate, or discontinue your participation, you are free to do so at any time.
 **RISKS**
 The risks and discomfort associated with participation in this study are no greater than those ordinarily encountered in daily life or during other online activities. The survey will mainly ask about your attitudes toward climate change and energy production.
 **BENEFITS** There are no direct benefits for your participation

**COMPENSATION** You will receive a base compensation of £9.00/h for taking part in this study. All compensation will be delivered to you via the Prolific automatic Completion Code.

**DATA PROTECTION** Your individual privacy will be maintained in all published and written data resulting from this study. You have been assigned a personal anonymous identifier which will be translated at the study entry into an anonymous code, and the data will be collected, transferred, or stored using only this anonymous code. Any emails exchanged between participants (i.e. you) and the researchers will also be deleted within six months of the experiment completion. The anonymous data will be stored on the institutional computer of Tobia Spampatti, access to which is protected by a password. Should you refuse to consent, or should you revoke your consent at any time during the experiment, all data collected will be deleted at the conclusion of the experiment. The results of this research study may be presented in a cumulative matter at scientific or professional meetings or published in scientific journals. The data will not be used for commercial purposes. Data will be kept indefinitely (i.e. without a time limit). Anonymous data may be subject to re-use in future research and may be deposited on platforms for "open science" (collaborative research work tools) in order to share them with others researchers.

**ACCESS TO RESULTS** The results of this study contribute to academic research and you will have access to our published papers, as announced on our university websites and at the Consumer Decision and Sustainable Behavior Laboratory website (www.unige.ch/fapse/decisionlab/).

**CONTACT INFORMATION** If you have any questions or concerns about this study, you should feel free to ask them by contacting the principal investigator: Tobia Spampatti Consumer Decision and Sustainable Behavior Lab University of Geneva, Campus Biotech Chemin des Mines 9, 1202 Geneva-CH Email: tobia.spampatti@unige.ch

Consent **CONSENT** Based on the information presented above, I confirm that I wish to take part in the study and I authorize:

|  | Yes (23) | No (24) |
| --- | --- | --- |
| 1. The use of the data for scientific purpose, and the publication of the results of the study in scientific journals or books, given that the data will be anonymous and that no information about my identity will be disclosed. (1) |  |  |

End of Block: Consent

Start of Block: No consent

Unconsented As you do not wish to participate in this study, please return your submission to Prolific by selecting the "Stopped without completing" button.

End of Block: No consent

Start of Block: Demographics

Instructions_demo
Thank you very much for agreeing to take part to the study.

 We will begin with some questions about yourself, your opinion about climate change, and a small mental exercise.

| Page Break |  |
| --- | --- |

Gender Which gender do you identify with?

- Male (0)
- Female (1)
- Non-binary / third gender (2)
- Prefer not to say (999)

Age How old are you (please write the number)?

________________________________________________________________

Education How many years of formal education have you completed?

- 0-6 (up to grade school/elementary school) (1)
- 7-12 (up to high school) (2)
- 13-16 (college/undergraduate university/certificate training) (3)
- More than 17 years (doctorate degree, medical degree, etc.) (4)
- Prefer not to answer (999)

S_media Do you have an active social media account?

- No (-1)
- Yes (1)

Pol_ideo What is your political orientation for the issues listed below? 
Please note, by "liberal" we mean classically left-wing, and by "conservative", we mean classically right-wing.

|  | Extremely liberal/left-wing | Moderate | Extremely conservative/right-wing | Prefer not to respond |
| --- | --- | --- | --- | --- |

|  | 0 | 10 | 20 | 30 | 40 | 50 | 60 | 70 | 80 | 90 | 100 |
| --- | --- | --- | --- | --- | --- | --- | --- | --- | --- | --- | --- |

| **For social issues** (e.g., health care, education, etc.) (1) |  |
| --- | --- |
| **For economic issues** (e.g., taxes) (9) |  |

Trust_science How much do you trust scientists to do what is right for your country?

- A lot (4)
- Some (3)
- Not too much (2)
- Not at all (1)

End of Block: Demographics

Start of Block: Soft_attention

Soft_attention For this question, please select **3** to make sure you are paying attention

- 1 (1)
- 2 (2)
- 3 (3)
- 4 (4)
- 5 (5)

End of Block: Soft_attention

Start of Block: Penalty

Penalty_time Timing

First Click (1)

Last Click (2)

Page Submit (3)

Click Count (4)

Display This Question:

If Soft_attention ! = 3

Penalty
Ups!

This text is being presented to you because you have unfortunately failed the attention check. No harm done, but

 **Please pay more attention to the experiment!**

 If you fail the next attention check, you will have to be redirected out of the survey.

 You will now have to repeat the previous question, please answer carefully this time.

End of Block: Penalty

Start of Block: Hard_Attention

Hard_attention For this question, please select **3** to make sure you are paying attention

- 1 (1)
- 2 (2)
- 3 (3)
- 4 (4)
- 5 (5)

End of Block: Hard_Attention

Start of Block: CRT

CRT_1 How many cubic feet of dirt are there in a hole that is 3’deep x 3’ wide x 3’ long? (Please provide the answer in numbers)

________________________________________________________________

CRT_2 A farmer had 15 sheep and all but 8 died. How many are left? (Please provide the answer in numbers)

________________________________________________________________

CRT_3 Emily’s father has three daughters. The first two are named April and May. What is the third daughter’s name?

________________________________________________________________

CRT_4 If you’re running a race and you pass the person in second place, what place are you in?

________________________________________________________________

End of Block: CRT

Start of Block: SASSY

SASSY_imp How important is the issue of global warming to you personally?

- Extremely important (1)
- Very important (2)
- Moderately important (3)
- Slightly important (4)
- Not at all important (5)

SASSY_worry How worried are you about global warming?

- Very worried (1)
- Somewhat worried (2)
- Not very worried (3)
- Not at all worried (4)

SASSY_self How much do you think global warming will harm you personally?

- A great deal (1)
- A moderate amount (2)
- Only a little (3)
- Not at all (4)
- Don't know (5)

SASSY_others How much do you think global warming will harm you future generations of people?

- A great deal (1)
- A moderate amount (2)
- Only a little (3)
- Not at all (4)
- Don't know (5)

End of Block: SASSY

Start of Block: Instructions

Instructions
*Y*ou will be presented with a series statements from actual social media posts about climate change/global warming. There are **10** in total.

 We are interested in your opinion about the following:
 1) Do you think that the statement is likely to be true?
 2) Is the statement more favorable for liberals or conservatives?
 3) Is the statement important?
 4) Is the statement provoking emotions?
 5) Have you ever seen or heard about the news story before?
 6) Would you consider sharing the statement on social media?

 Please note: some of the statements contain acronyms, for global warming (GW-AGW), United Nations (UN), the Intergovernmental Panel on Climate Change (IPCC), and the UNFCCC Conference of the Parties (COP).

 Please click the blue arrow to begin

End of Block: Instructions

Start of Block: Misinfo_1

Misinfo_1


 ${lm://Field/1}

 **#### Due to the nature of the data, the climate disinformation statements are hidden from view here but can be requested at:** [**https://www.swissubase.ch/en/share/btRbxspvu-ZL5lPOlPRVinxfDeZAZ2PzrCHBX3shc55Z3lR_x3v7It-t_beZrMKfJH8**](https://www.swissubase.ch/en/share/btRbxspvu-ZL5lPOlPRVinxfDeZAZ2PzrCHBX3shc55Z3lR_x3v7It-t_beZrMKfJH8) **####**

Misinfo_Timing Timing

First Click (1)

Last Click (2)

Page Submit (3)

Click Count (4)

| Page Break |  |
| --- | --- |

affect_action How does this statement make you feel about climate mitigation action?

- [1] very negatively (1)
- [2] (2)
- [3] (3)
- [4] (4)
- [5] (5)
- [6] (6)
- [7] (7)
- [8] (8)
- [9] (9)
- [10] very positively (10)

affect_science How does this statement make you feel about climate science?

- [1] very negatively (1)
- [2] (2)
- [3] (3)
- [4] (4)
- [5] (5)
- [6] (6)
- [7] (7)
- [8] (8)
- [9] (9)
- [10] very positively (10)

share If you were to see the above statement on social media, how likely would you be to share it?

- Extremely unlikely (1)
- Moderately unlikely (2)
- Slightly unlikely (3)
- Slightly likely (4)
- Moderately likely (5)
- Extremely likely (6)

true What is the likelihood that the above statement is true?

- Extremely unlikely (1)
- Moderately unlikely (2)
- Slightly unlikely (3)
- Slightly likely (4)
- Moderately likely (5)
- Extremely likely (6)

slant How favorable would it be to liberals versus conservatives?

- More favorable for liberals (1)
- Moderately more favorable for liberals (2)
- Slightly more favorable for liberals (3)
- Slightly more favorable for conservatives (4)
- Moderately more favorable for conservatives (5)
- More favorable for conservatives (6)

importance Assuming the statement is entirely accurate, how important would this statement be?

- Extremely unimportant (1)
- Moderately unimportant (2)
- Slightly unimportant (3)
- Slightly important (4)
- Moderately important (5)
- Extremely important (6)

manipulative How manipulative do you find this statement?

- [1] Not at all manipulative (1)
- [2] (2)
- [3] (3)
- [4] (4)
- [5] (5)
- [6] (6)
- [7] Very manipulative (7)

persuasive How persuasive do you find this statement?

- [1] Not at all persuasive (1)
- [2] (2)
- [3] (3)
- [4] (4)
- [5] (5)
- [6] (6)
- [7] Very persuasive (7)

trustworthy How trustworthy do you find this statement?

- [1] Not at all trustworthy (1)
- [2] (2)
- [3] (3)
- [4] (4)
- [5] (5)
- [6] (6)
- [7] Very trustworthy (7)

|  |  |
| --- | --- |

emotions How ... do you find this statement?

|  | Not at all (1) | Slightly (2) | Somewhat (3) | Moderately (4) | Very much (5) | Extremely (6) |
| --- | --- | --- | --- | --- | --- | --- |
| Exciting (excite) |  |  |  |  |  |  |
| Worrying (worry) |  |  |  |  |  |  |
| Funny (funny) |  |  |  |  |  |  |
| Informative (informative) |  |  |  |  |  |  |

anger To what extent is this statement outrage provoking?

- Not at all (1)
- Slightly (2)
- Somewhat (3)
- Moderately (4)
- Very much (5)
- Extremely (6)

familiarity Are you familiar with the above statement (have you seen or heard about it before)?

- Yes (1)
- Unsure (0)
- No (-1)

End of Block: Misinfo_1

Start of Block: Debriefing

Instructions_Timing Timing

First Click (1)

Last Click (2)

Page Submit (3)

Click Count (4)

Debriefing
Thank you very much for participating in this study!
The aim of this study was to understand **how misinformation about climate change/global warming spread.** 

 That’s right, the statements you read were false information about the science of climate change/global warming, and manipulative statements meant to delay urgent climate action!
 Please remember that, regardless of the posts you have read, **over** 99% of climate scientists agree **that climate change is real and it is humanly caused by greenhouse gas emissions due to the burning of fossil fuels and we have to implement urgent actions to avoid the worst of it**. The IPCC report, the most authoritative body of climate change evidence, concluded that “It is unequivocal that human influence has warmed the atmosphere, ocean and land. Widespread and rapid changes in the atmosphere, ocean, cryosphere and biosphere have occurred.” If you encounter statements that doubt the existence of human-caused climate change in the future, even from Nobel laureates, remember that **climate science is now settled, and that climate change is real and humanly caused**. With this study, we hope to develop ways to fight misinformation about climate change spread by vested interests.

Debriefing
If you want to read more about the IPCC report and the best scientific evidence we have so far on the state of our climate and how it is changing, click here.

*Thank you for your participation, please click the arrow to end the experiment and be redirected to the panel provider’s website.*

Comment If you want to leave a comment, you can do so here:

________________________________________________________________

End of Block: Debriefing

**References**

Coan, T. G., Boussalis, C., Cook, J., & Nanko, M. O. (2021). Computer-assisted classification of contrarian claims about climate change. *Scientific Reports*, *11*(1), 22320. <https://doi.org/10.1038/s41598-021-01714-4>

Lamb, W. F., Mattioli, G., Levi, S., Roberts, J. T., Capstick, S., Creutzig, F., Minx, J. C., Müller-Hansen, F., Culhane, T., & Steinberger, J. K. (2020). Discourses of climate delay. *Global Sustainability*, *3*, e17. <https://doi.org/10.1017/sus.2020.13>

Rahmani Azad, Z., Spampatti, T., Günther, A., Hahnel, U.J.J., (in preparation). *Unraveling the threads: Analyzing Twitter Discourses on Climate Action and Policy Support*.

Zanartu, F., Otmakhova, Y., Cook, J., & Frermann, L. (2024). Generative Debunking of Climate Misinformation. Preprint retrieved at: <https://doi.org/10.48550/arXiv.2407.05599>
